# Size Sensitivity of Metabolite Diffusion in Macromolecular Crowds

**DOI:** 10.1021/acs.nanolett.3c05100

**Published:** 2024-04-12

**Authors:** Edyta Raczyłło, Dariusz Gołowicz, Tomasz Skóra, Krzysztof Kazimierczuk, Svyatoslav Kondrat

**Affiliations:** † Institute of Physical Chemistry, Polish Academy of Sciences, 01-224 Warsaw, Poland; ‡ Department of Theoretical Chemistry, Institute of Chemical Sciences, Faculty of Chemistry, Maria Curie-Skłodowska University in Lublin, 20-031 Lublin, Poland; § Scientific Computing and Imaging Institute, University of Utah, Salt Lake City, Utah 84112, United States; ∥ Centre of New Technologies, University of Warsaw, 02-097 Warsaw, Poland; ⊥ Institute for Computational Physics, University of Stuttgart 70569, Stuttgart, Germany

**Keywords:** macromolecular crowding, intracellular diffusion, size dependence, metabolites, Stokes−Sutherland−Einstein relation, nanoviscosity

## Abstract

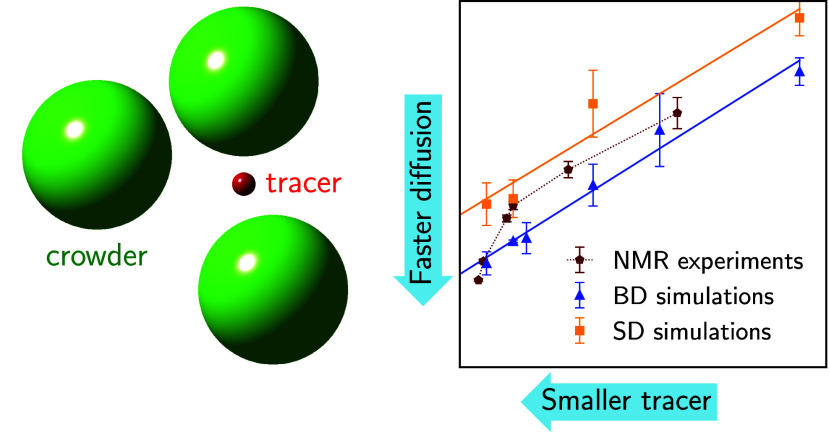

Metabolites play crucial roles in cellular processes, yet their diffusion in the densely packed interiors of cells remains poorly understood, compounded by conflicting reports in existing studies. Here, we employ pulsed-gradient stimulated-echo NMR and Brownian/Stokesian dynamics simulations to elucidate the behavior of nano- and subnanometer-sized tracers in crowded environments. Using Ficoll as a crowder, we observe a linear decrease in tracer diffusivity with increasing occupied volume fraction, persisting—somewhat surprisingly—up to volume fractions of 30–40%. While simulations suggest a linear correlation between diffusivity slowdown and particle size, experimental findings hint at a more intricate relationship, possibly influenced by Ficoll’s porosity. Simulations and numerical calculations of tracer diffusivity in the *E. coli* cytoplasm show a nonlinear yet monotonic diffusion slowdown with particle size. We discuss our results in the context of nanoviscosity and discrepancies with existing studies.

Macromolecules crowd the intracellular space of living cells. In *E. coli*, for example, they occupy up to 44% of the cell interior.^1−3^ Such macromolecular crowding^4−10^ has diverse effects on physicochemical processes: It shifts equilibria^2,11−13^ and cooperativity^14^ of chemical reactions, can inhibit or enhance enzyme-catalyzed reactions,^15−18^ and affects metabolic channeling,^19−21^ gene regulation,^22,23^ and cell growth.^24^ Yet, the most apparent and extensively studied but not fully understood effect is the slowdown of intracellular diffusion. Studies of diffusion in crowded environments have mainly been limited to the diffusion of macromolecules,^25−33^ while the diffusion of metabolites, smaller, subnanometer-sized molecules, transformed by enzymes in various biochemical pathways received much less attention. Understanding metabolite diffusion in crowded environments is, however, vital as it determines the rates of diffusion-limited reactions,^34−36^ affecting metabolism and signal transduction,^37,38^ microbial dynamics,^39,40^ etc. For instance, studies^41−44^ have indicated that the diffusivity of metabolites in brain cells can change due to pathology, providing potentially useful medical information.

García-Pérez et al.^45^ used pulsed-field gradient spin–echo ^1^H NMR to investigate metabolite diffusion inside living cells and in solutions of synthetic crowders. They found a diffusion slowdown of about 50% compared to zero crowding, irrespective of metabolite size. Dauty and Verkman^46^ employed fluorescence correlation spectroscopy (FCS) and reported that Ficoll crowding reduced the diffusion of small solutes and macromolecules to a similar extent. However, more recent studies have indicated that crowding slows the diffusion of smaller particles to a lesser extent.^26,47,48^ This result agrees with the conclusion drawn from an isotope-filtered pulsed-gradient stimulated-echo (PGSTE) NMR study by Rothe et al.^49^ but contrasts with large-scale molecular dynamics simulations^50^ reporting a substantially stronger slowdown of metabolite diffusion compared to macromolecules.

The diffusion coefficient (*D*) is related to the particle size via the Stokes–Sutherland–Einstein (SSE) equation^51,52^
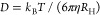
1where η is the fluid dynamic viscosity; *R*_H_ is the particle hydrodynamic radius; *k*_B_ is the Boltzmann constant; and *T* is the absolute temperature. This relation works excellently for dilute systems, but there has yet to be a consensus on whether it holds for crowded environments^32,47^ and at the nanoscale.^53^ Some^28,54−56^ but not all^46,57^ diffusion measurements performed in polymer-crowded solutions indicate the breakdown of the SSE equation. Moreover, some authors reported a stronger slowdown of the diffusion of larger particles in vivo than predicted by the SSE equation,^58−63^ sometimes called a sieving effect,^28,63^ while others^27,32,64,65^ did not, except for particles of masses larger than 100 kDa (*R*_H_ ≈ 4.7 nm utilizing conversion from refs (66 and 67)). Several studies^47,56^ generalized eq 1 by introducing a size-dependent “nanoviscosity” η(*R*_H_), which interpolates between the macroviscosity experienced by a macroscopically large object (*R*_H_ ≫ *R*_c_) and the solvent viscosity η_0_ for *R*_H_ ≪ *R*_c_, where *R*_c_ is the crowder radius. Thus, η → η_0_ for *R*_H_/*R*_c_ → 0, independently of the crowder concentration, implying *D* → *D*_0_ with *D*_0_ being the diffusion coefficient at zero crowding, which is not aligned with other studies.^45,46^

In this Letter, we discuss how macromolecular crowding affects the diffusion of metabolites and its size dependence. We utilized PGSTE-NMR to measure the diffusivity of various particles under crowding conditions generated by Ficoll. NMR offers an advantage over frequently used fluorescence-based methods as it does not require labeling, which can disturb tracer diffusion, an especially pertinent concern for the small tracers examined in this study. To interpret our experimental results, we employed two computational approaches: Brownian dynamics (BD) simulations, in which we neglect hydrodynamic interactions (HI), and Stokesian dynamics (SD) simulations, which incorporate HI. While atomistic molecular dynamics simulations are increasingly employed for modeling such systems,^7^ we deliberately opt for coarse-grained simulations to focus on generic effects unobscured by chemical complexity and a variety of interparticle interactions. Our choice of inert tracers and crowders in experiments aligns with this simulation approach. However, we stress that investigating the influence of chemical interactions and molecular structures represents an essential next step toward a more comprehensive understanding of metabolite diffusion in intracellular environments.

## Diffusion of Metabolites from NMR Experiments

The fundamental concept underlying NMR diffusion measurements involves the decay of spin coherence resulting from diffusion during a pulsed-gradient spin echo. To ascertain the diffusion coefficient, a range of spectra with varying pulsed-field gradient strength or echo duration are acquired, and the decay of peaks is analyzed using the Stejskal–Tanner equation.^68^ We conducted PGSTE-NMR experiments for particles ranging in size from *R*_H_ ≈ 0.28 to *R*_H_ ≈ 3.30 nm, as estimated with the SSE equation based on the PGSTE-NMR data obtained for the corresponding D_2_O solutions at 298 K (Table S1). The resulting hydrodynamic radii differ less than 8% compared to previously reported data.^69−74^

We generated crowding using Ficoll PM70 (Ficol70) at various concentrations ranging from zero (no crowding) to a concentration of 1.2 mM, corresponding to an occupied volume fraction of ϕ_occ_ ≈ 40% (Table S1). We estimated ϕ_occ_ using the hydrodynamic radius *R*_c_ = 5.1 nm, as provided by Cytiva and used in several publications.^28,29^ Other studies have reported slightly different values of the hydrodynamic radius^75^ and observed polydispersity^28^ and bimodality^76^ of Ficoll. Such subtleties can influence ϕ_occ_ and, therefore, the rate of diffusion slowdown. However, we anticipate that these factors would impact our results quantitatively rather than qualitatively, maintaining the fundamental dependence on tracer sizes.

We prepared samples with concentrations of tracer molecules between 0.1 and 1 mM (Table S1). This concentration range allowed us to obtain sufficiently good signal-to-noise ratios in PGSTE-NMR data, simultaneously minimizing the crowding effects due to the tracers. All measurements were performed on the 700 MHz Agilent NMR spectrometer at 298 K using a Bipolar Pulse Pair Stimulated Echo pulse sequence (Section S1). Figure 1a illustrates ^1^H PGSTE-NMR spectra for alanine, phenylalanine, and cyanocobalamin at 10% crowding (for other spectra, see Figures S3–S31). For all studied samples, we could identify a peak or a group of peaks, well separated from Ficoll’s signals, and calculate diffusion coefficients based on their integrals.^77^

**Figure 1:**
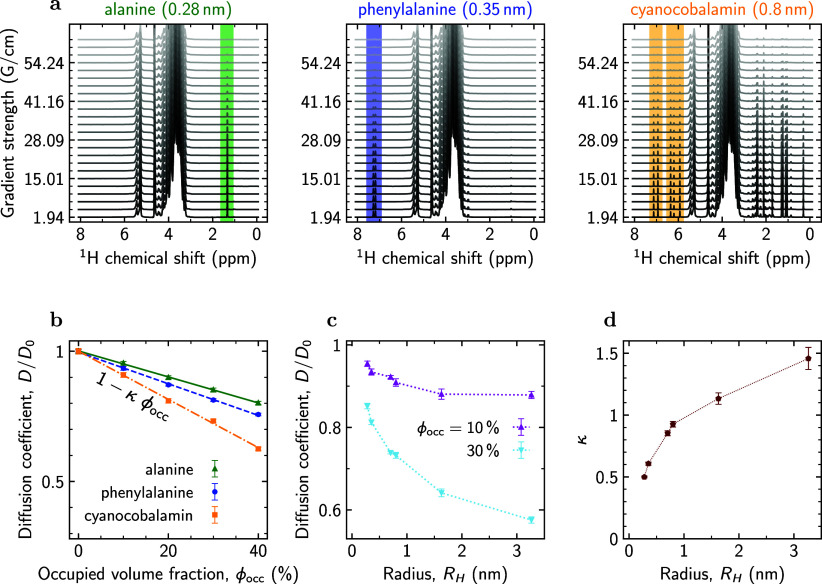
NMR results. (a) Examples of ^1^H PGSTE-NMR spectra series for alanine (left), phenylalanine (middle), and cyanocobalamin (right) in a Ficoll70 solution with 10% occupied volume fraction (for a pure Ficoll70 ^1^H NMR spectrum, see Figure S2). The shaded spectral regions show well-resolved peaks of alanine (green), phenylalanine (blue), and cyanocobalamin (yellow) selected for calculating self-diffusion coefficients. The ^1^H PGSTE-NMR spectra measured for α-cyclodextrin (hydrodynamic radius *R*_H_ ≈ 0.70 nm), ubiquitin (*R*_H_ ≈ 1.63 nm), and hemoglobin (*R*_H_ ≈ 3.27 nm) at different Ficoll’s concentrations can be found in Figures S3–S31. (b) Relative diffusion coefficient (*D*/*D*_0_) of alanine (*R*_H_ ≈ 0.28 nm), phenylalanine (*R*_H_ ≈ 0.35 nm), and cyanocobalamin (*R*_H_ ≈ 0.80 nm) as functions of the occupied volume fraction ϕ_occ_ of Ficoll70 (see Table S1 for details). *D*_0_ is the corresponding diffusion coefficient in the absence of crowders. Results for other particles are presented in Figure S38. Signal attenuation plots together with their fits are shown in Figures S32–S37 for all studied samples. The lines show results of fitting the PGSTE-NMR data with eq 2. (c) *D*/*D*_0_ as a function of particle size for two values of the occupied volume fraction ϕ_occ_. (d) Parameter κ (defined by eq 2) as a function of particle size.

The SSE relation, eq 1, with a size-independent viscosity implies that the ratio *D*/*D*_0_ does not depend on *R*_H_. However, Figure 1b shows *D*/*D*_0_ for alanine (*R*_H_ ≈ 0.28 nm), phenylalanine (*R*_H_ ≈ 0.35 nm), and cyanocobalamin (*R*_H_ ≈ 0.80 nm) versus ϕ_occ_, revealing a more pronounced diffusion slowdown for larger particles at all ϕ_occ_. This behavior is shown explicitly in Figure 1c for two values of ϕ_occ_, demonstrating a monotonic dependence of *D*/*D*_0_ on *R*_H_ across a broad range of *R*_H_ values.

Moreover, Figure 1b shows a linear decrease in the diffusivity with ϕ_occ_. We observed this linear dependence also for larger particles (Figure S38), which suggests the possibility of describing our experimental data through a linear fit
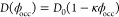
2where ϕ_occ_ is expressed in decimals and κ is a fitting parameter, characterizing diffusion slowdown independently of ϕ_occ_. (Previous work^45,78,79^ used symbol α instead of κ in eq 2. Since α is conventionally reserved for denoting the exponent of anomalous diffusion,^80^ we have chosen a different symbol to avoid confusion.) Fitting results for κ are presented in Figure 1d, showing that κ behaves monotonically but nonlinearly with *R*_H_.

## Results of BD Simulations

BD simulations are a convenient technique for studying particle dynamics in the diffusive regime. In these simulations, the solvent is treated implicitly via stochastic Brownian forces and friction, while inertia is disregarded. Due to their typically larger time steps and the implicit solvent treatment, BD simulations are computationally less demanding than atomistic molecular dynamics simulations,^7,50^ enabling longer simulation times, essential for studying long-time diffusion phenomena. We conducted BD simulations without HI and addressed hydrodynamic effects separately.

**Figure 2:**
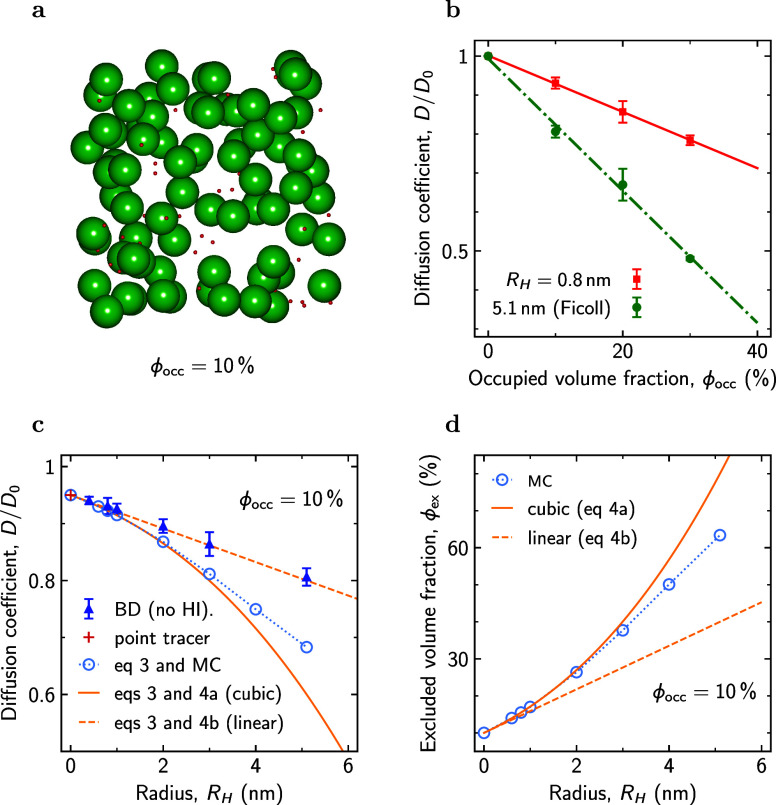
Results of BD simulations. (a) Snapshot from BD simulations of a mixture of metabolites (hydrodynamic radius *R*_H_ = 0.8 nm) and Ficoll70 (*R*_H_ = 5.1 nm). The total occupied volume fraction ϕ_occ_ ≈ 10%. Simulations have been carried out without hydrodynamic interactions (HI). (b) Relative diffusion coefficient (*D*/*D*_0_) of the metabolites and Ficoll70 as a function of ϕ_occ_. *D*_0_ is the corresponding diffusion coefficient in infinite dilution (ϕ_occ_ = 0) computed according to the SSE formula (eq 1). (c) *D*/*D*_0_ vs hydrodynamic radius *R*_H_ for ϕ_occ_ = 10%. The triangles show the BD simulation results. The result for a point tracer was obtained with the Maxwell–Garnett formula^82^ (eq 2 with κ = 1/2). The circles show the diffusion coefficient calculated using eq 3 with ϕ_ex_ computed by the MC method. Results of using the linear (eq 4b) and cubic (eq 4a) approximations for ϕ_ex_ are shown by the dashed and solid lines, respectively. (d) Excluded volume fraction ϕ_ex_ vs *R*_H_ obtained by the MC method (symbols) and by using eqs 4a and 4b. Occupied volume fraction ϕ_occ_ = 10%. Excluded volume fractions for other values of ϕ_occ_ are shown in Figure S39.

Simulations were performed for a metabolite/Ficoll70 mixture (Figure 2a) across various values of ϕ_occ_ and *R*_H_. Ficoll70 and metabolites were modeled as hard spheres with a radius of *R*_c_ = 5.1 nm and of various radii *R*_H_, respectively. We included 50 metabolites (ca. 0.2 mM) in all simulation systems to gather sufficient statistics (Table S2). Simulations were carried out with the pyBrown package^81^ using the forward Euler propagation scheme (Section S2.1).

Figure 2b shows that the relative diffusion coefficient (*D*/*D*_0_) of both Ficoll and metabolites decreases linearly with ϕ_occ_, consistent with our experimental results. In Figure 2c, we plot *D*/*D*_0_ versus *R*_H_ for ϕ_occ_ = 10%, revealing a linear decrease of *D*/*D*_0_ with *R*_H_. For a point particle (*R*_H_ → 0), the diffusion coefficient can be calculated analytically using the Maxwell–Garnett formula,^82^ which amounts to setting κ = 1/2 in eq 2.^83−86^ For ϕ_occ_ = 10%, this yields *D*/*D*_0_ = 0.95 (the red plus in Figure 2c), providing a reasonable extrapolation of the simulation data.

One can use the Maxwell–Garnett formula to estimate the *R*_H_ dependence of *D* by considering diffusion of a point particle in a sea of crowders enlarged by the tracer radius, i.e., of radius *R*_c_ + *R*_H_. This procedure replaces ϕ_occ_ in the Maxwell–Garnett formula with the excluded volume fraction, ϕ_ex_, giving
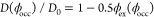
3For a point particle (*R*_H_ → 0), ϕ_ex_ → ϕ_occ_. To calculate ϕ_ex_ for nonpoint particles, we used the Monte Carlo (MC) method, which relies on the iterative insertion of a tracer into a crowded system and computing the percentage of unsuccessful insertions occurring due to overlaps with the crowders. We sampled crowder configurations with BD simulations and averaged the results over five distinct configurations (Section S3). In Figure 2d, we plot ϕ_ex_ vs *R*_H_ computed with the MC method for ϕ_occ_ = 10% and compare it with two approximate expressions:
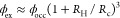
4a
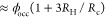
4bEquation 4a follows from summing up excluded volumes generated by all crowders present in the system, neglecting excluded volume overlaps (valid only at low ϕ_occ_). The second expression (eq 4b) is a linear approximation of eq 4a. Equation 4b shows decent agreement with the MC data for small *R*_H_ but deviates progressively as the particle size increases. Nevertheless, using this approximation one easily arrives at eq 2 with κ(*R*_H_) = 0.5(1 + 3*R*_H_/*R*_c_). Interestingly, for *R*_H_ = *R*_c_, i.e., for self-crowding, this equation predicts κ = 2, in agreement with the theoretical result by Hanna et al.^79^ for the same system. Figure 2c shows that the linear approximation provides excellent agreement with BD simulations over the whole range of *R*_H_ up to *R*_H_ = *R*_c_. However, this agreement appears to be coincidental. Using the cubic expression (eq 4a) and the MC data for ϕ_ex_ in eq 3 leads to a stronger slowdown of tracer diffusivity than that obtained by BD simulations (Figure 2c). This discrepancy likely arises due to crowder mobility, which enhances tracer diffusivity^87,88^ but is not considered in the Maxwell–Garnett formula. Nevertheless, it captures the *R*_H_/*R*_c_ → 0 limit, which is reasonable considering that the diffusivity of a point particle is infinitely high compared to that of crowders.

## Effect of Hydrodynamic Interactions (HI)

When a particle moves in a fluid, it induces fluid flow that influences the motion of other particles and vice versa. These indirect forces among particles are referred to as HI.^89,90^ HI can be effectively incorporated in BD simulations by appropriately adjusting the position-dependent diffusion tensor. Here, we utilize the diffusion tensor of the F-version Stokesian dynamics (SD), which encompasses many-body far-field and near-field (lubrication) hydrodynamics. This approach considers particle translations and forces while disregarding rotations and torques.^89,90^ Such a simplification aligns with the coarse-grained models of spherical crowders utilized in our study. We conducted SD simulations of Ficoll70–metabolite mixtures using the pyBrown simulation package,^81^ employing the midpoint propagation scheme (Section S2.2).

**Figure 3:**
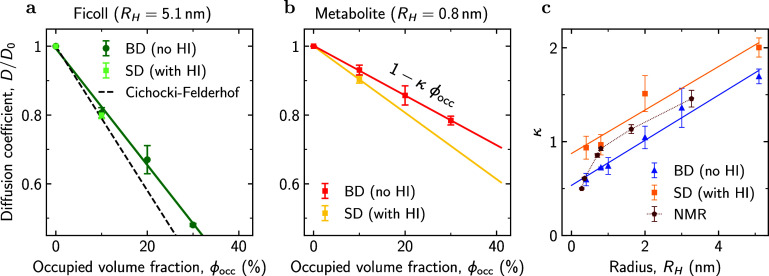
Effect of hydrodynamic interactions (HI). Comparison of relative diffusion coefficients (*D*/*D*_0_) as functions of an occupied volume fraction (ϕ_occ_) for (a) Ficoll70 and (b) metabolites in Ficoll70–metabolite mixtures simulated with and without HI. The symbols show the simulation results, and the solid lines are the linear fits. The dashed line in (a) shows the theoretical result by Cichocki and Felderhof.^78^ Simulations without HI were performed using the Brownian dynamics (BD) method (see Figure 2). Simulations with HI were carried out using the F-version Stokesian dynamics (SD). The time-dependent diffusion coefficients are shown in Figure S40. (c) Parameter κ (defined by eq 2) as a function of tracer radius *R*_H_. The filled pentagons show the PGSTE-NMR results from Figure 1d.

Figure 3a presents relative diffusion coefficients (*D*/*D*_0_) of Ficoll70 obtained using SD simulations and BD simulations without HI. For comparison, we also show the analytical result by Cichocki and Felderhof^78^ for self-crowding, demonstrating a decent agreement with our simulations and suggesting that the presence of metabolites (at concentrations below 0.2 mM) has a vanishing effect on the Ficoll diffusion.

In Figure 3b, we plot the *D*/*D*_0_ for metabolites. Comparing this figure with Figure 3a reveals that HI have a stronger impact on metabolite diffusion than on Ficoll70 diffusion, resulting in a more significant slowdown compared to systems without HI. This is likely because the motion of metabolites is hydrodynamically correlated with the motion of slower Ficoll70, which enhances the slowdown of the metabolite diffusion. This tendency is also reflected in parameter κ (Figure 3c). Notably, however, both BD and SD simulations predict qualitatively similar dependence on tracer size.

For comparison, Figure 3c also shows our PGSTE-NMR results. For large tracers (*R*_H_ ≳1 nm), the decrease of κ with decreasing *R*_H_ aligns with the trends observed in BD and SD simulations. For smaller tracers, however, the κ values are significantly lower, indicating faster diffusion than predicted by simulations. While we cannot pinpoint the exact reason for this diffusivity enhancement, we note studies^76,91^ reporting Ficoll’s porosity on the subnanometer scale. In terms of excluded volume, this subnanoscale porosity is not visible to large tracers (larger than a typical pore size) but could provide a larger accessible space for subnanosized particles, thus enhancing their long-time diffusivity. Given Ficoll’s common use as a model crowder, further studies on these issues are crucial and can bring valuable insights into macromolecular crowding and metabolite diffusion in systems with polymeric crowders.

## Metabolite Diffusion in the Cytoplasm

**Figure 4:**
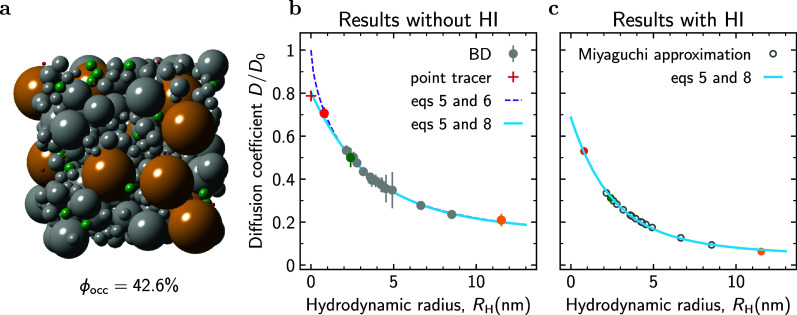
Diffusion in the *E. coli* cytoplasm. (a) Snapshot from BD simulations of metabolites (red spheres) in the *E. coli* cytoplasm model of refs (26 and 92). The green and orange spheres represent green fluorescent protein (GFP) and ribosomes, respectively, and the remaining macromolecules are shown in gray. (b) Relative diffusion coefficients (*D*/*D*_0_) vs hydrodynamic radius (*R*_H_) of various macromolecules of the *E. coli* cytoplasm, additionally containing 50 metabolites of radius *R*_H_ = 0.8 nm. The results were obtained by BD simulations without hydrodynamic interactions (HI). The red plus shows the result for a point tracer obtained using the Maxwell–Garnett formula (eq 2 with κ = 1/2) with an occupied volume fraction of ϕ_occ_ = 0.426, corresponding to the cytoplasm. The dashed line shows the results of fitting the simulation data by eqs 5 and 6. The solid line shows the fitting by eq 5 with *a* = 1 and eq 8 for *R*_eff_. (c) Effect of HI on diffusion in the *E. coli* cytoplasm. The open circles show results for *D*/*D*_0_ obtained by applying the Miyaguchi approach.^30^ The blue solid line shows results of fitting the numerical data with eqs 5 and 8. The red, green, and orange circles in panels (b) and (c) highlight the results for metabolites, GFP, and ribosome, respectively (see panel (a)).

Thus far, our discussion has focused on crowding generated by macromolecules of the same size. However, biological fluids, such as the cytoplasm, consist of macromolecules with diverse shapes and sizes. To explore the impact of such polydispersity on metabolite diffusion, we adopted a model proposed by Ridgway et al.^92^ This model features particles with sizes and concentrations representative of the *E. coli* cytoplasm. Simulations by Ando and Skolnick^26^ on atomistic and coarse-grained versions of this model yielded similar results for particle diffusivity. We thus chose the computationally more efficient coarse-grained *E. coli* model, including additionally 50 metabolites of radius *R*_H_ = 0.8 nm (Figure 4a and Table S3). Due to the computational intensity of SD simulations, we employed BD simulations without HI.

Figure 4b shows the size dependency of relative diffusivity in the cytoplasm, revealing a notably milder deceleration of metabolite diffusion compared to that of macromolecules. The diffusion slowdown systematically escalates with increasing particle size and, unlike for monodisperse crowding, exhibits a nonlinear behavior.

To investigate the impact of HI, we followed an approximate approach developed by Miyaguchi.^30^ Miyaguchi computed the far-field mobility functions using the twin-multipole expansion^93^ up to the order 1/*r*^100^ (where *r* is the particle separation) and estimated the relative diffusivity employing the Batchelor method^94^ (Section S4). The calculations reveal that the tracer diffusivity exhibits a qualitatively similar size dependence as in the absence of HI, but with a more pronounced slowdown (Figure 4c, see also Figure S1).

We endeavored to predict the diffusion slowdown for metabolites in the cytoplasm using eq 2 and our results for monodisperse crowding (Section S6). While feasible in the absence of HI, the presence of HI rendered this procedure unviable. This observation accentuates the crucial role of crowding polydispersity and HI in metabolite diffusion.

## Nanoviscosity

Kalwarczyk et al.^47^ have proposed a phenomenological equation for size-dependent “nanoviscosity” η(*R*_H_), gaining popularity in analyzing experimental data, particularly for biologically relevant fluids such as the cytoplasm.^47,67,95−97^ This equation relates η(*R*_H_) and solvent viscosity η_0_ or, alternatively, the diffusion coefficients in crowded and dilute systems by^47,98^
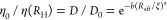
5where ξ characterizes the structural properties of a crowded medium and can be interpreted as the intercrowder gap; *R*_eff_ is an effective hydrodynamic radius (see below); *a* is an exponent; and *b* a factor, both of the order of unity.^47^ Kalwarczyk et al.^47^ related *R*_eff_ to the tracer radius and an effective crowder radius *R*_c_ by
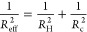
6*R*_eff_ approaches the crowder radius for large tracers (*R*_H_ ≫ *R*_c_) and the tracer radius for small tracers (*R*_H_ ≪ *R*_c_), interpolating η(*R*_H_) between macroscale and nanoscale viscosities, respectively.^47^

For a hard-sphere system, Kalwarczyk et al.^98^ proposed ξ = *R*_*g*_ψ_rcp_(1 – ϕ_occ_)/ϕ_occ_, where ψ_rcp_ ≈ 1.76 is ϕ_occ_/(1 – ϕ_occ_) evaluated at the random close packing (ϕ_occ_ ≈ 0.638) and *R*_g_ is the crowder’s gyration radius (we took *R*_g_ = *R*_c_ as in our simulations). We used this equation for ξ combined with eqs 5 and 6 to fit the simulation data for the cytoplasm (Figure 4c) and obtained *b* ≈ 3.7 ± 0.2, *a* ≈ 0.67 ± 0.02, and *R*_c_ ≈ (12.1±1.6) nm. Figure 3c shows that eqs 5 and 6 provide a good fit for intermediate and large *R*_H_, but they do not capture the behavior of metabolites. Indeed, in the *R*_H_/*R*_c_ → 0 limit, eq 6 gives *R*_eff_ ≈ *R*_H_. Considering metabolites with *R*_H_ ≪ ξ, we expand eq 5 to obtain , which gives a nonlinear dependence on ϕ_occ_ and *R*_H_ in their lowest order, *viz*.
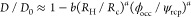
7unlike eq 2. Equation 7 shows explicitly that *D* → *D*_0_ as *R*_H_/*R*_c_ → 0 independently of ϕ_occ_. This is not consistent with our experiments and simulations, nor with the findings in other works.^45,46^

Equations 5 and 6 can be modified to reproduce the small *R*_H_ and ϕ_occ_ behaviors by introducing a step-like *R*_H_-dependent exponent *a*(*R*_H_) (such that *a* is unity for *R*_H_/*R*_c_ → 0 and constant when *R*_H_ ≫ *R*_c_) and a “minimal length” *R*_min_ in *R*_eff_ to prevent *R*_eff_ ≈ *R*_H_ → 0 when *R*_H_ → 0 (point particle). As an example, one can take
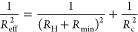
8It is not difficult to see that this extension leads to eq 2 with κ depending linearly on *R*_H_ in the *R*_H_ → 0 limit. Since we lack data for macroscopically large tracers, we set *a* = 1 in fitting our results for the cytoplasm with eqs 5 and 8 (Table S4). Figure 4 shows that this fit decently reproduces the *R*_H_ → 0 behavior. It is worth noting, however, that we encountered difficulties fitting the results of the SD simulations of Ando and Skolnick^26^ (Section S5), which emphasizes the necessity for further studies assessing the applicability and significance of these empirical equations.

## Conclusions

We have investigated metabolite diffusion in intracellular-like macromolecularly crowded environments. For monodisperse crowding with Ficoll70, our experiments and simulations revealed a linear dependence of the relative tracer diffusivity on the occupied volume fraction (ϕ_occ_). Notably, this linearity persisted up to an ϕ_occ_ as high as 30–40% (Figures 1b and 2b), suggesting the practicality of using the decay coefficient κ in eq 2 as a convenient parameter characterizing the extent of diffusion slowdown in such crowded environments. It will be beneficial to explore this dependence for crowders and tracers of different shapes and featuring chemical interactions.

Our study revealed a consistent decrease in relative tracer diffusivity, particularly represented by the slowdown parameter κ, with increasing tracer size. We observed it with NMR experiments (Figure 1b–d) as well as with BD (Figure 2c) and SD (Figure 3c) simulations under monodisperse crowding conditions. A similar pattern was found for the *E. coli* cytoplasm characterized by crowder size polydispersity (Figure 4). While these results align with some simulations^26^ and FCS studies^47^ (albeit with quantitative differences), they contrast with earlier NMR^45^ and other FCS^32,46^ investigations reporting no size dependence.

In a recent FCS study, for instance, Bellotto et al.^32^ found a size-independent diffusion slowdown (ca. 80%) within the *E. coli* cytoplasm for particles of sizes ranging from 26.9 kDa to 100 kDa (2.8 to 4.7 nm). Our calculations for the cytoplasm revealed the slowdown of a similar magnitude (see Figure 4c for *R*_H_ ≲5 nm) but with noticeable size dependency. NMR experiments and simulations for Ficoll crowding at physiologically relevant concentrations displayed a size-dependent slowdown, but of a weaker magnitude, possibly due to the monodisperse nature of crowding (see Figure 1c for ϕ_occ_ = 30%). Given the alignment of our experiments and simulations for particles larger than 1 nm (Figure 3c), the prediction of size-dependent diffusion slowdown in the cytoplasm shown in Figure 4 suggests that any observed independence on tracer size^32^ is not generic but may result from the intricate interplay between crowding polydispersity and interparticle interactions. Despite extensive studies,^27,29−33,48,50,99,100^ further experimental and theoretical work is required for a more comprehensive understanding of these effects. Investigations employing molecular dynamics simulations^7,50^ and in vivo NMR spectroscopy^101^ hold particular promise in providing deeper microscopic insights into intracellular diffusion processes, and we hope our findings motivate such endeavors.

## Supporting Information Available

Details of NMR experiments, BD and SD simulations, MC calculations, the Miyaguchi approximation, a fitting procedure, and a comparison of monodisperse and polydisperse crowding. Supplementary tables including the ingredients and selected properties of the studied samples, the simulated systems, the composition of the *E. coli* cytoplasm model, and the fitting results. Supplementary plots showing NMR spectra for Ficoll70 and all tracers; all signal attenuation plots; diffusion coefficients as functions of ϕ_occ_ for α-cyclodextrin, ubiquitin, and hemoglobin; excluded volume vs. tracer radius; and time-dependent diffusivities from SD simulations of Ficoll–metabolite mixtures and BD simulations of the *E. coli* cytoplasm model (PDF)
