# Supplementary material for: Size Sensitivity of Metabolite Diffusion in Macromolecular Crowds
Source: Nano Lett. 2024 Apr 12;24(16):4801–9. doi: 10.1021/acs.nanolett.3c05100 (PMC11057039; doi:10.1021/acs.nanolett.3c05100)
Supplement: Supplementary file 1 — nl3c05100_si_001.pdf [file nl3c05100_si_001.pdf]

Supplementary Material to "Size Sensitivity of Metabolite Diffusion in Macromolecular Crowds"

Edyta Raczyłło,[†,‡,⊥] Dariusz Gołowicz,[†,⊥] Tomasz Skóra,[†,¶] Krzysztof Kazimierczuk,[§] and Svyatoslav Kondrat[*,†,‖]

†*Institute of Physical Chemistry, Polish Academy of Sciences, 01-224 Warsaw, Poland*

‡*Department of Theoretical Chemistry, Institute of Chemical Sciences, Faculty of Chemistry, Maria Curie-Skłodowska University in Lublin, 20-031 Lublin, Poland*

¶*Scientific Computing and Imaging Institute, University of Utah, Salt Lake City, Utah 84112, United States*

§*Centre of New Technologies, University of Warsaw, 02-097 Warsaw, Poland*

‖*Institute for Computational Physics, University of Stuttgart, Stuttgart, Germany*

⊥*Contributed equally to this work.*

E-mail: skondrat@ichf.edu.pl;svyatoslav.kondrat@gmail.com

Contents

S1 Details of NMR measurements **S3**

S1.1 Sample preparation S3

S1.2 NMR experiments S3

S1.3 NMR spectra S6

S2 Simulations **S7**

S2.1 Brownian dynamics S7

S2.2 Stokesian dynamics S8

S2.3 Trajectory analysis S10

S2.4 Simulated systems S10

S3 Monte Carlo integration of excluded volumes **S13**

S4 Miyaguchi approximation for diffusion slowdown **S14**

S5 Fitting tracer diffusivity in the cytoplasm **S16**

S6 Monodisperse *vs.* polydisperse crowding **S17**

S7 Supplementary figures **S18**

References **S42**

S1 Details of NMR measurements

S1.1 Sample preparation

We investigated the effect of macromolecular crowding induced by Ficoll on the following molecules separately: phenylalanine, alanine, cyanocobalamin, α-cyclodextrin, ubiquitin, and hemoglobin.

We prepared one aqueous sample without crowder for each tracer molecule listed above and three or four aqueous samples with linearly increasing Ficoll concentrations. Samples containing only the tracer were prepared by diluting a premade stock solution (D_2O) of the alanine (Sigma-Aldrich) or phenylalanine (Sigma-Aldrich) or cyanocobalamin (POL-AURA) with D_2O (99.9%, Eurisotop) or by dissolving a weighted amount of α-cyclodextrin (Sigma-Aldrich) or ubiquitin (from bovine erythrocytes, Sigma-Aldrich) or hemoglobin (from bovine erythrocytes, Sigma-Aldrich) in D_2O. The samples containing tracer and crowder were prepared either by mixing a tracer's stock solution (alanine, phenylalanine, cyanocobalamin) with proper aliquots of a premade 1.4 mM stock Ficoll (Sigma-Aldrich) solution and D_2O or by dissolving weighted tracer amount (α-cyclodextrin, ubiquitin, hemoglobin) in Ficoll's stock solution further diluted with D_2O. Due to potential aggregation issues, we did not use stock solutions of α-cyclodextrin, ubiquitin, and hemoglobin.

A complete list of prepared samples and their concentrations can be found in Table S1.

S1.2 NMR experiments

We carried out all NMR measurements using an Agilent DirectDrive2 spectrometer equipped with a room-temperature HCN probe, operating at a frequency of 700 MHz for ^1H nuclei. The temperature of each sample was set to 298 K. The pulse sequence employed for measuring diffusion coefficients was Bipolar Pulse Pair Stimulated Echo (Dbppste), available in VnmrJ 4.2 software.

Before the measurement of metabolite samples, we mapped (z-) pulsed-field gradient

Table S1: **Table showing ingredients and selected properties of the studied samples.** The left-hand side grouped column (ϕ_{occ}) shows volume fractions occupied by tracer molecules (Trc): phenylalanine (Phe), alanine (Ala), cyanocobalamin (Cya), $\alpha-$cyclodextrin (α-Cyc), ubiquitin (Ubi), hemoglobin (Hem), and crowder: Ficoll (Fic). Column C_m shows the corresponding molar concentration of the ingredients (we calculated Ficoll's molar concentration using its average molecular weight of 70 kDa). Column D includes self-diffusion coefficients for tracer molecules obtained from ^{1}H PGSTE-NMR experiments. The uncertainties were determined from the standard errors obtained from the covariance matrix during the fitting of the PGSTE-NMR data to equation (S1). The right-hand side column R_H shows the approximate hydrodynamic radius calculated for tracer molecules in the samples without crowder using Stokes-Einstein-Sutherland equation (Eq. (1); $\eta = 1.096\,35\,\mathrm{cP}$,[1] $T = 298\,\mathrm{K}$).

	ϕ_{occ} (%)							C_m(mM)		D (nm²/µs)	R_H (nm)
Sample	Phe	Ala	Cya	α-Cyc	Ubi	Hem	Fic	Fic	Trc	Trc	
1	0.011	-	-	-	-	-	-	-	1.00	562.2 ± 2.3	0.354 ± 0.001
2	0.011	-	-	-	-	-	10.039	0.30	1.00	525.3 ± 3.5	-
3	0.011	-	-	-	-	-	20.077	0.60	1.00	489.8 ± 2.0	-
4	0.011	-	-	-	-	-	30.116	0.90	1.00	456.9 ± 2.6	-
5	0.011	-	-	-	-	-	40.154	1.20	1.00	425.7 ± 2.3	-
6	-	0.006	-	-	-	-	-	-	1.01	712.7 ± 3.4	0.280 ± 0.001
7	-	0.006	-	-	-	-	10.039	0.30	1.01	680.2 ± 3.2	-
8	-	0.006	-	-	-	-	20.077	0.60	1.01	640.9 ± 3.6	-
9	-	0.006	-	-	-	-	30.116	0.90	1.01	607.1 ± 3.8	-
10	-	0.006	-	-	-	-	40.154	1.20	1.01	571.4 ± 3.1	-
11	-	-	0.063	-	-	-	-	-	0.51	249.2 ± 1.9	0.800 ± 0.006
12	-	-	0.063	-	-	-	10.039	0.30	0.51	226.5 ± 1.5	-
13	-	-	0.063	-	-	-	20.077	0.60	0.51	201.7 ± 1.5	-
14	-	-	0.063	-	-	-	30.116	0.90	0.51	182.5 ± 1.1	-
15	-	-	0.063	-	-	-	40.154	1.20	0.51	155.8 ± 1.2	-
16	-	-	-	0.045	-	-	-	-	0.53	282.9 ± 0.8	0.704 ± 0.002
17	-	-	-	0.042	-	-	10.039	0.30	0.50	260.9 ± 0.8	-
18	-	-	-	0.040	-	-	20.077	0.60	0.46	234.4 ± 1.0	-
19	-	-	-	0.047	-	-	30.116	0.90	0.55	209.0 ± 0.6	-
20	-	-	-	0.042	-	-	40.154	1.20	0.50	188.2 ± 0.6	-
21	-	-	-	-	0.097	-	-	-	0.09	122.4 ± 1.3	1.628 ± 0.017
22	-	-	-	-	0.116	-	10.039	0.30	0.10	107.8 ± 1.0	-
23	-	-	-	-	0.105	-	20.077	0.60	0.09	91.7 ± 0.9	-
24	-	-	-	-	0.095	-	30.116	0.90	0.08	78.5 ± 0.8	-
25	-	-	-	-	0.095	-	40.154	1.20	0.08	67.7 ± 0.5	-
26	-	-	-	-	-	1.003	-	-	0.12	61.0 ± 0.4	3.265 ± 0.023
27	-	-	-	-	-	0.986	10.039	0.30	0.11	53.6 ± 0.4	-
28	-	-	-	-	-	1.032	20.077	0.60	0.12	42.4 ± 0.4	-
29	-	-	-	-	-	1.043	30.116	0.90	0.12	35.1 ± 0.4	-

uniformity,[2] calibrated gradient strength using the known diffusion coefficient of residual HDO in the D_2O sample at 298K,[3] and performed other standard calibrations (pulse width, shims). We set critical parameters Δ (diffusion delay) and δ (gradient duration) for the NMR diffusion experiments as follows: $\Delta = 50\,\text{ms}$, $\delta = 2\,\text{ms}$ for all phenylalanine and alanine samples; $\Delta = 125\,\text{ms}$, $\delta = 2\,\text{ms}$ for all cyanocobalamin and $\alpha-$cyclodextrin samples; $\Delta = 100\,\text{ms}$, $\delta = 3\,\text{ms}$ for all ubiquitin samples; $\Delta = 135\,\text{ms}$, $\delta = 3\,\text{ms}$ for all hemoglobin samples. We ran a ^{1}H Dbppste pulse sequence with a 24-step gradient strength array ranging from 1.94 G/cm to 62.08 G/cm for all samples. The data were collected by averaging 16 scans for each gradient step with the following acquisition parameters: 15.9 ppm spectral width, 2.936 s acquisition time, and 5 s relaxation delay. We preprocessed data using Python's nmrglue library.[4] The preprocessing included exponential signal weighting (lb=2 Hz), zero-filling to 262,144 data points, and linear baseline correction with the manually set node points for the regions of interest. To determine the self-diffusion coefficients, we fitted a decay of the selected peaks with a modified Stejskal–Tanner equation that accounts for non-uniform gradients (NUG)[2,5] using Python's SciPy library.[6]

$$S(g) = S(0) + e^{-\sum_{n=1}^{4} c_n (D\gamma^2\delta^2\Delta' g^2)^n}, \tag{S1}$$

where S is the stimulated echo signal amplitude, g is the gradient amplitude, D is the self-diffusion coefficient, γ is the magnetogyric ratio (^{1}H), δ is the total duration of the positive and negative gradient pulses forming a single bipolar gradient, c are the probehead-specific coefficients, and $\Delta' = \Delta - \delta/3 - \tau/2$ (τ is the delay between positive and negative gradient pulses).

We observed mono-exponential decays for the studied tracers.

S1.3 NMR spectra

We include ^{1}H NMR spectrum of pure Ficoll (Fig. S2) and all ^{1}H PGSTE-NMR spectra collected in our study (Fig. S3-S31). Also, we include signal attenuation plots (Fig. S32-S37) used for calculating diffusion coefficients by fitting Eq. (S1) to the corresponding data.

S2 Simulations

S2.1 Brownian dynamics

We performed Brownian dynamics (BD)[7–9] simulations with `pyBrown` software,[10] using forward Euler propagation scheme:

$$\boldsymbol{r}(t + \Delta t) = \boldsymbol{r}(t) + \sqrt{2\boldsymbol{D}\Delta t}\boldsymbol{X}, \tag{S2}$$

where $\boldsymbol{r}$ is $3N$-dimensional position vector, t is time, Δt is propagation step, $\boldsymbol{X}$ is standard white-noise vector, and $\sqrt{\boldsymbol{D}}$ is a diagonal matrix with entries being square roots of particles' diffusion coefficients, which are in turn computed from the Stokes-Einstein-Sutherland equation, *i.e.*:

$$\sqrt{\boldsymbol{D}_{ij}} = \sqrt{\frac{k_B T}{6\pi\eta R_{\mathrm{H}}}}\boldsymbol{I}\delta_{ij}. \tag{S3}$$

Here, k_B is Boltzmann constant, T is temperature, η is fluid dynamic viscosity, and R_{H} is hydrodynamic radius of the spherical particle.

We placed the simulated particles in cubic boxes of $75\,\mathrm{nm} \times 75\,\mathrm{nm} \times 75\,\mathrm{nm}$ and applied periodic boundary conditions in all directions. We accounted for hard-sphere repulsion by rejecting simulation steps leading to overlaps and repeating them to the point, when they do not do so.

We used propagation step $\Delta t = 0.1\,\mathrm{ps}$, an order of magnitude smaller than in our previous papers[11–14] to account for smaller sizes, and thus larger Brownian leaps, of the metabolites. Our simulations lasted for at least $7\,\mathrm{\mu s}$. We set $T = 298.15\,\mathrm{K}$ and $\eta = 1.02\,\mathrm{cP}$.

S2.2 Stokesian dynamics

We performed F-version Stokesian dynamics (SD)[15–18] simulations with `pyBrown` software[10] as well. In contrast to a diagonal diffusion matrix (Eq. (S3)), and popular RPY tensor,[19–21] SD representation of diffusion matrix is not sourceless, *i.e.*, $\nabla \cdot \boldsymbol{D} \neq \boldsymbol{0}$. To account for divergence term in original formulation of BD,[7] we used midpoint propagation scheme,[22] *i.e.*:

$$\boldsymbol{r}(t+\Delta t) \;=\; \boldsymbol{r}(t) + \sqrt{2\Delta t}\boldsymbol{B}(t)\boldsymbol{X} + \frac{m}{2}\sqrt{2\Delta t}\left[\boldsymbol{D}\left(t+\frac{\Delta t}{m}\right)\left(\boldsymbol{B}(t)^T\right)^{-1} - \boldsymbol{B}(t)\right]\boldsymbol{X}. \quad \text{(S4)}$$

Here, $\boldsymbol{D}$ is no longer diagonal, and $\boldsymbol{B}$ is its Choleski factor: $\boldsymbol{B}^T\boldsymbol{B} = \boldsymbol{D}$. We used $m = 100$, following Ando and Skolnick.[23]

The overall scheme of diffusion matrix construction may be summarized as follows:

1. compute generalized RPY diffusion matrix:[19–21]

$$\boldsymbol{D}_{ij}(r_{ij}) = \frac{k_B T}{8\pi\eta r_{ij}}\left[\left(1+\frac{R_{\mathrm{H},i}^2+R_{\mathrm{H},j}^2}{3r_{ij}^2}\right)\boldsymbol{I} + \left(1-\frac{R_{\mathrm{H},i}^2+R_{\mathrm{H},j}^2}{r_{ij}^2}\right)\hat{\boldsymbol{r}}_{ij}\hat{\boldsymbol{r}}_{ij}^T\right] \quad \text{(S5a)}$$

   for $r_{ij} > R_{\mathrm{H},i} + R_{\mathrm{H},j}$ and

$$\boldsymbol{D}_{ij}(r_{ij}) = \frac{k_B T}{8\pi\eta r_{ij}}\left[\frac{16r_{ij}^3(R_{\mathrm{H},i}+R_{\mathrm{H},j}) - [(R_{\mathrm{H},i}-R_{\mathrm{H},j})^2+3r_{ij}^2]^2}{32r_{ij}^3}\boldsymbol{I} + \frac{3[(R_{\mathrm{H},i}-R_{\mathrm{H},j})^2-r_{ij}^2]^2}{32r_{ij}^3}\hat{\boldsymbol{r}}_{ij}\hat{\boldsymbol{r}}_{ij}^T\right] \quad \text{(S5b)}$$

   for $R_{\mathrm{H},ij}^M - R_{\mathrm{H},ij}^m < r_{ij} < R_{\mathrm{H},i} + R_{\mathrm{H},j}$, where $R_{\mathrm{H},ij}^M = \max(R_{\mathrm{H},i}, R_{\mathrm{H},j})$ and $R_{\mathrm{H},ij}^m = \min(R_{\mathrm{H},i}, R_{\mathrm{H},j})$. Finally:

$$\boldsymbol{D}_{ij} = \frac{k_B T}{6\pi\eta R_{\mathrm{H},ij}^M}\boldsymbol{I}, \quad \text{(S5c)}$$

   for $r_{ij} < R_{\mathrm{H},ij}^M - R_{\mathrm{H},ij}^m$.

2. perform Ewald summation of generalized RPY matrix.[24]

3. compute far-field N-body resistance matrix $\boldsymbol{\xi}_{ff}^{NB}$ by inverting Ewald-summated generalized RPT diffusion matrix:

$$\boldsymbol{\xi}_{ff}^{NB} = k_B T \boldsymbol{D}_{\mathrm{RPY}}^{-1} \tag{S6}$$

4. for each pair of particles compute a translational part of 2-body resistance matrix $\boldsymbol{\xi}^{2B}$ following expressions derived by Jeffrey and Onishi[25] and a translational part of 2-body far-field resistance matrix $\boldsymbol{\xi}_{ff}^{2B}$ by inverting 2-body mobility matrix in RPY approximation.

5. for each pair of particles, form $3N \times 3N$ matrix with zeros everywhere apart from blocks ii, ij, ji and jj, for which substitute $\boldsymbol{\xi}^{2B} - \boldsymbol{\xi}_{ff}^{2B}$ for ij pair and sum all these matrices, forming lubrication correction matrix $\Delta\boldsymbol{\xi}_{nf,0}$

6. following Cichocki *et al.*,[26] transform the lubrication correction in a following way:

$$\Delta\boldsymbol{\xi}_{nf} = q\Delta\boldsymbol{\xi}_{nf,0}q^T; \tag{S7a}$$

where:

$$q = \frac{1}{2}\begin{bmatrix} \boldsymbol{I} & -\boldsymbol{I} \\ -\boldsymbol{I} & \boldsymbol{I} \end{bmatrix}. \tag{S7b}$$

to exclude the collective motion and avoid divergence of the diffusion matrix.

7. the final lubrication corrected diffusion matrix is

$$\boldsymbol{D} = k_B T \left[\boldsymbol{\xi}_{ff}^{NB} + \Delta\boldsymbol{\xi}_{nf}\right]^{-1}. \tag{S8}$$

Detailed expressions can be found in appendices B-C of ref. 27.

In Ewald summation, we used cutoffs for real and reciprocal space contributions of $m = n = 2$, respectively, and the convergence-regulating coefficient $\alpha = \sqrt{\pi}$. For short-range hydrodynamic interactions, we set a cutoff of $2(r_{ij} - R_{\mathrm{H},i} - R_{\mathrm{H},j})/(R_{\mathrm{H},i} + R_{\mathrm{H},j})$. For computational efficiency, we followed refs. 11–13,23, and performed time-consuming computation of RPY matrix every 100 steps. However, we computed lubrication correction and Choleski decomposition of final diffusion matrix every step, as the near-field hydrodynamic interactions at short distances are changing much more quickly than the far-field ones.

S2.3 Trajectory analysis

We computed diffusion coefficients from time-averaged mean squared displacement (TAMSD):

$$\mathrm{TAMSD}(m\Delta t) = \frac{1}{N_{\mathrm{traj}}} \sum_{i=1}^{N_{\mathrm{traj}}} \frac{1}{N_{\mathrm{steps}} - m} \sum_{k=0}^{N_{\mathrm{steps}}-m-1} \{\boldsymbol{r}_i\,[(k+m)\Delta t] - \boldsymbol{r}_i\,[k\Delta t]\}^T \{\boldsymbol{r}_i\,[(k+m)\Delta t] - \boldsymbol{r}_i\,[k\Delta t]\}, \tag{S9}$$

where Δt is analysis window length, N_{traj} is number of trajectories for a given system, N_{steps} is number of steps in a given trajectory and $\boldsymbol{r}$ is position vector of i-th particle. We used window length equal to 15 ns.

The long-time diffusion coefficients D_l is the average value of D/D_0 estimated between 3 µs and 5 µs. In order to obtain uncertainty of the D_l we divided all trajectories into 5 subsets and computed standard deviations of the mean.

S2.4 Simulated systems

We have performed BD simulations for crowding with Ficoll70 using several occupied volume fractions ϕ_{occ} and tracer sizes. All systems are listed in Table S2. In addition, we performed SD simulations for $\phi_{\mathrm{occ}} = 10\,\%$ and tracers of sizes $R_{\mathrm{H}} = 0.4\,\mathrm{nm}$, $R_{\mathrm{H}} = 0.8\,\mathrm{nm}$, and $R_{\mathrm{H}} =$

0.8 nm.

We also performed BD simulations (without hydrodynamic interactions) for the *E. coli* cytoplasm model of Ridgway *et al.*[28] and Ando and Skolnick.[23] The cytoplasm system additionally contained 50 metabolites of the hydrodynamic radius $R_{\mathrm{H}} = 0.8\,\mathrm{nm}$. The details are shown in Table S3.

Table S2: Composition of mixtures of metabolites and Ficoll70 ($R_{\mathrm{H}} = 5.1\,\mathrm{nm}$). The highlighted rows show the systems used in SD simulations.

Volume fraction	Hydrodynamic radius	Molecule number		Molar fraction	
	metabolites	metabolites	Ficoll70	metabolites	Ficoll70
10.01 %	0.4 nm	50	76	0.40	0.60
10.04 %	0.8 nm	50	76	0.40	0.60
20.05 %	0.8 nm	50	152	0.25	0.75
30.06 %	0.8 nm	50	228	0.18	0.82
10.06 %	1.0 nm	50	76	0.40	0.60
10.01 %	2.0 nm	50	73	0.41	0.59
10.03 %	3.0 nm	50	66	0.43	0.57

Table S3: **Constituents of the cytoplasm model and their abundances.** The model is taken from ref. 23. R_{H} stands for hydrodynamic (Stokes) radius. The box volume is set to $V = (75\mathrm{nm})^3$, and the total macromolecular concentration amounts to $250\,\mathrm{mg/L}$

**Compound**		**Composition**
name	R_{H} (nm)	Number of particles
70S Ribosome	11.52	10
GroEL/ES	8.52	13
RNA polymerase	6.65	26
Phosphoglycerate dehydrogenase	4.92	4
Cystathionine gamma-synthase	4.54	2
Glyceraldehyde-3-P dehydrogenase	4.31	15
Ribonuclease HI	2.14	24
Green Fluorescent Protein	2.40	39
Malonyl CoA-acyl carrier protein transacylase	2.57	33
Triosephosphate isomerase	3.17	44
Fructose 1-6 bisphosphate aldolase	3.68	32
Enolase	3.59	32
6-phosphogluconate dehydrogenase	3.92	11
Phosphoenolpyruvate-protein phosphotransferase	4.25	12
Initial tRNA	2.78	126
Metabolite	0.8	50

S3 Monte Carlo integration of excluded volumes

To calculate volumes excluded by crowders in particular systems to the selected tracers, ϕ_{ex}, we used Monte Carlo integration implemented in our custom `ExVol` package (`https://tskora.github.io/ExVol/`). The algorithm is summarized below.

For a given crowded system, we load a configuration of its constituents from a selected timestep of BD simulation. Then, a chosen tracer particle is inserted into that configuration at a position drawn from a uniform random distribution. If the insertion leads to an overlap of the tracer with any of the crowders, we increment a counting variable n (initially set to 0) by 1; we do not increment n otherwise. We remove the tracer and repeat the insertion multiple times. After very large number of insertions, N, the volume excluded to the chosen tracer is estimated as:

$$\phi_{\mathrm{ex}} = \frac{n}{N}. \tag{S10}$$

This quantity is related to the free energy of inserting the tracer to the crowded system in a selected configuration.[29]

We averaged such obtained quantity over multiple configurations of the crowded system, *i.e.*, we repeated the procedure described above for other timesteps from the BD trajectory. We set N to 100000 insertions and used 5 configurations for each crowded system.

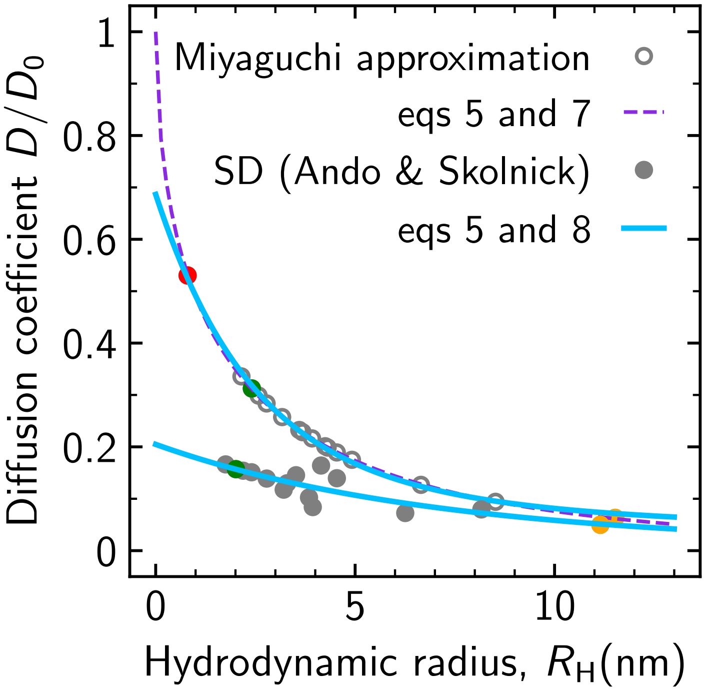

Figure S1: **Diffusion in the *E. coli* cytoplasm.** The open circles show the results obtained by applying the Miyaguchi[30] approach. The filled circles show the SD simulation results by Ando and Skolnick for the same cytoplasm model but without metabolites.[23] The red, green, and orange circles highlight results for the metabolites, GFP, and ribosome, respectively (Fig. 4). The dash line shows the results of fitting the simulation data by Eqs. (5) and (6) of the main text. Fitting the SD results with these equations did not yield any reasonable outcome. The blue solid lines show the results of fitting the numerical data with Eqs. (5) and (8) of the main text.

S4 Miyaguchi approximation for diffusion slowdown

We have employed the method developed by Miyaguchi[30] to study hydrodynamic effects on diffusion in the *E. coli* cytoplasm. According to this method, the diffusion coefficient of i'th particle is given by

$$D_i \approx D_{i,0}(1 - K_i \phi_{\mathrm{occ}}), \tag{S11}$$

where

$$K_i = K_i^{(S)} + K_i^{(I)} + K_i^{(B)}, \tag{S12}$$

Here $K_i^{(S)}$, $K_i^{(I)}$, and $K_i^{(B)}$ are the mobility coefficients describing short-time diffusivity, interactions (zero in our case), and the Brownian force, respectively. The equations for the mobility coefficients are lengthy and are not presented here (see ‘ref. 30). Computing them involves calculations of the mobility functions in the diffusion tensor and a steady-state solution of the corresponding Smoluchowski equation. The latter was done using the twin-multipole expansion of the Stokes equation to the order of $1/r^{100}$, where r is particle separation, and numerically solving the resulting recursion relations.[31] The former was computed as a series expansion including 99 terms.

For the *E. coli* cytoplasm, we used a model proposed by Ridgway *et al.*[28] and Ando and Skolnick,[23] adding additionally fifty metabolites of radius 0.8 nm (Table S3). All calculations of the mobility coefficients K_i for various particles of this cytoplasm model have been performed using the C++ code generously provided by Professor Miyaguchi.

Figure S1 compares the Miyaguchi approximation with the SD simulation results of Ando and Skolnick[23] for the same cytoplasm model but without metabolites. While the Miyaguchi approach provides a decent approximation for large macromolecules, it shows deviations for smaller particles, suggesting an even stronger slowdown of the metabolite diffusion.

Table S4: Results of fitting various results for the *E. coli* cytoplasm with Eq. (5) of the main text using the original[32] (Eq. (6) in the main text) and modified (Eq. (8) in the main text) expressions for R_{eff}. HI stands for hydrodynamic interactions.

Results	Expression for R_{eff}	a	b	R_{c} (nm)	R_{min} (nm)
BD (no HI)	Eq. (6)	0.67 ± 0.02	3.70 ± 0.17	12.1 ± 1.6	0
	Eq. (8)	1	4.80 ± 0.23	9.55 ± 0.70	1.04 ± 0.08
Miyaguchi app. (HI)	Eq. (6)	0.558 ± 0.003	19 ± 26	152 ± 377	0
	Eq. (8)	1	7.81 ± 0.28	9.42 ± 0.54	1.08 ± 0.06
SD[23] (with HI)	Eq. (6)	—	—	—	0
	Eq. (8)	1	18 ± 79	54 ± 295	11 ± 13

S5 Fitting tracer diffusivity in the cytoplasm

We have fitted our numerical results for tracer diffusivity within the *E. coli* cytoplasm with Eq. (5) using the original[33] (Eq. (6)) and modified (Eq. (8)) equations (all equation are from the main text). The fitting results are shown in Table S4.

While the fitting process was satisfactory for the results of BD simulations (without HI) and the Miyaguchi approximation, providing reasonable values for the fitting parameters, we encountered difficulties fitting the results of the SD simulations conducted by Ando and Skolnick.[23] Despite the fitting curve smoothly approximating the data (Fig. S1), we obtained inflated values of R_{min} and R_{c} accompanied by significant standard deviation errors (Table S4). Such inflated values may be related to the slowdown of short-time diffusivity due to near-field hydrodynamics present in SD simulations.[23] However, they can also result from the broad dispersion of the simulation data and the lack of data pertaining to macroscopically large particles. It is worth noting that attempting to fit the SD results with the original expression (Eqs. (5) and (6) of the main text) did not yield any viable outcome. Further studies are thus needed to assess the accuracy and significance of these empirical equations. Moreover, it would be useful to identify the fitting parameters with physically relevant properties through microscopic theory.

S6 Monodisperse *vs.* polydisperse crowding

Monodisperse crowding, employing synthetic crowders like Ficoll, is commonly used to study intracellular processes. How well does such crowding mimic the intracellular environment? Here, we analyse it in the context of metabolite diffusion using the results of our simulations for Ficoll-metabolite mixtures and the *E. coli* cytoplasm.

Employing Eq. (2) (see the main text) with $\kappa = 0.692$ (determined for the Ficoll70 crowder and the 0.8 nm metabolite from BD simulations without hydrodynamic interactions, see Fig. 2c in the main text) and setting $\phi_{\text{occ}} = 0.426$, corresponding to the *E. coli* cytoplasm, we estimated the metabolite diffusion coefficient to be $D/D_0 \approx 0.705$, in excellent agreement with the result of BD simulations for the cytoplasm ($D/D_0 \approx 0.71 \pm 0.03$). This agreement is likely due to comparable volumes excluded for a small tracer by monodisperse and polydisperse crowders (Fig. S39). In addition, owing to significant size differences, the mobility of crowders plays a less significant role for small tracers compared to macromolecular-sized tracers that are similar in size to the crowders.

From the SD simulations of the Ficoll-metabolite mixture, we found $\kappa \approx 0.966$ (Fig. 3c) yielding $D/D_0 \approx 0.59$ for the metabolite diffusion coefficient. This value is significantly higher than predicted by the Miyaguchi approximation ($D/D_0 \approx 0.53$). It is worth noting that this approximation likely underestimates the diffusion slowdown (Section S4). We therefore anticipate even larger differences between the results for monodisperse and polydisperse crowding.

Thus, while crowding polydispersity might be disregarded in the absence of hydrodynamic interactions, it evidently plays a significant role when such interactions are incorporated, even for small particles like metabolites.

S7 Supplementary figures

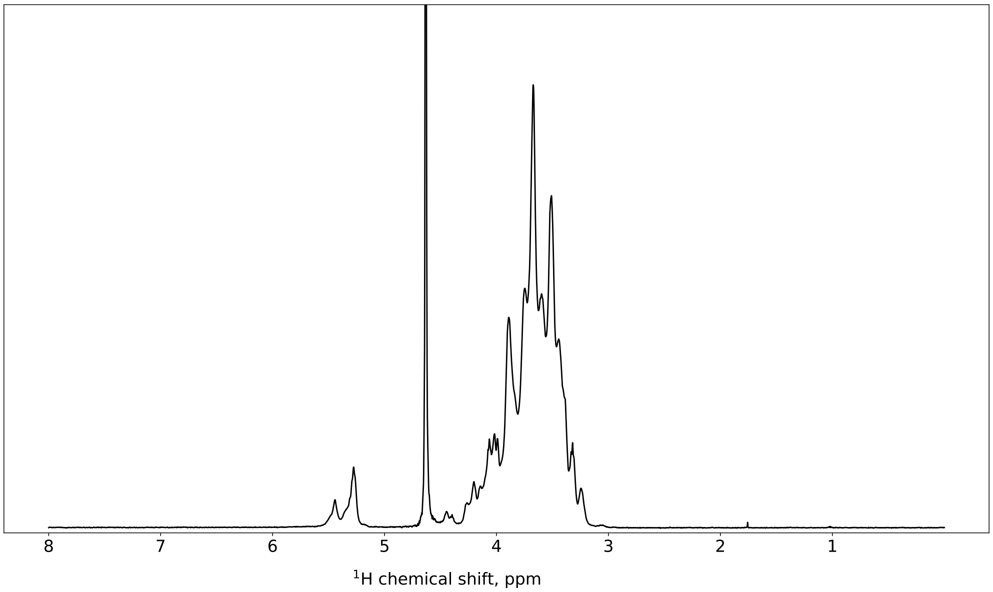

Figure S2: ^{1}H **NMR spectrum of Ficoll in D_2O** ($\phi_{occ} \approx 10\,\%$)

A note to all figures below showing NMR spectra: a region representing HOD peak has been removed to increase figures' clarity.

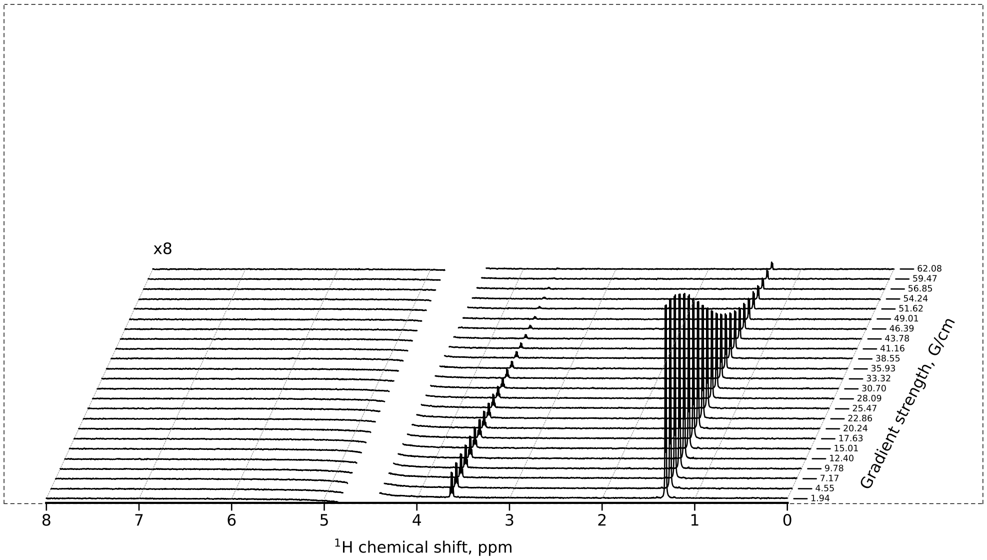

Figure S3: **Array of Dbppste ^{1}H NMR spectra of** $1.01\,\mathrm{mM}$ **alanine solution in D_2O as a function of diffusion-encoding gradient strength.** The intensity has been upscaled by a factor of eight.

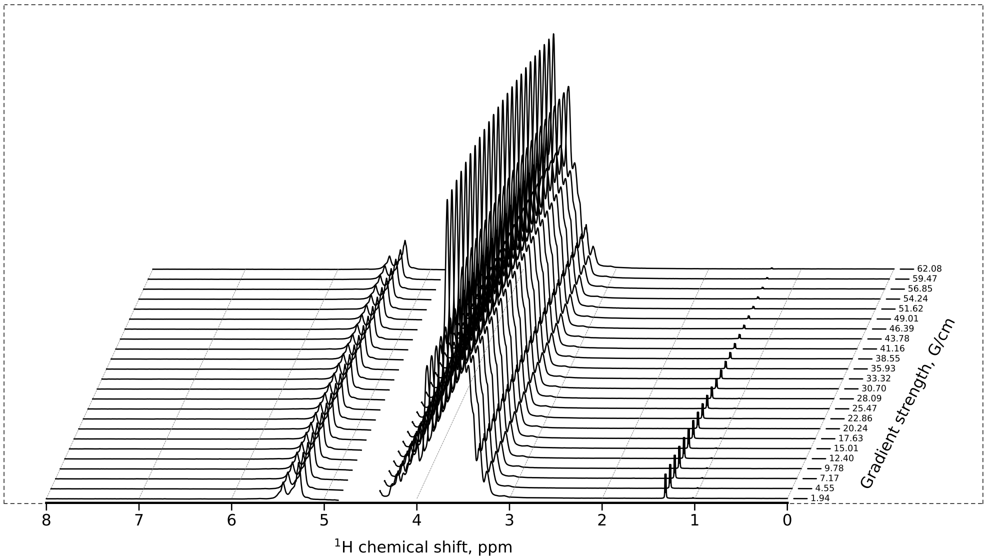

Figure S4: **Array of Dbppste ^{1}H NMR spectra of $1.01\,\mathrm{mM}$ alanine and Ficoll-PM70 ($\phi_{\mathrm{occ}}^{Ficoll} \approx 10\,\%$) mixture in $\mathbf{D_2O}$ as a function of diffusion-encoding gradient strength.**

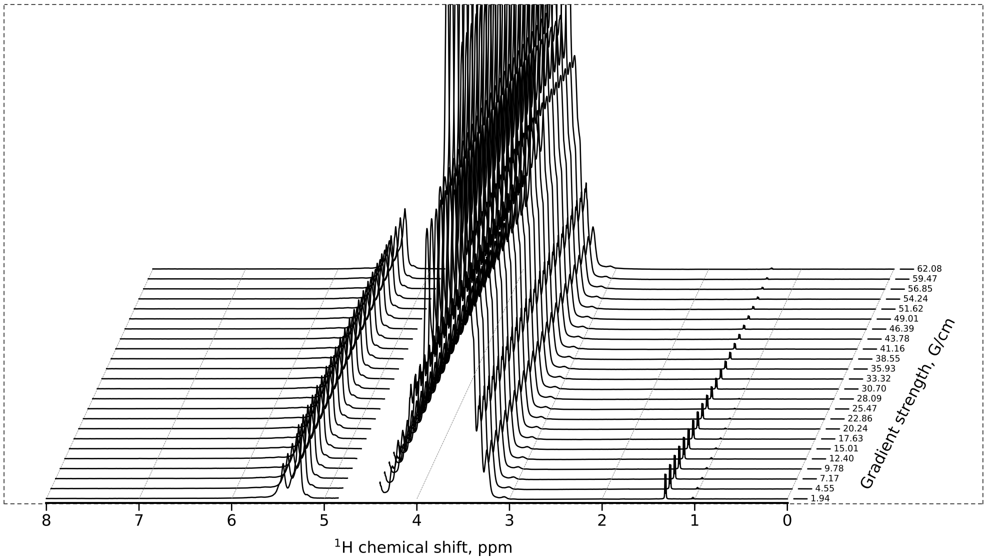

Figure S5: **Array of Dbppste ^{1}H NMR spectra of $1.01\,\mathrm{mM}$ alanine and Ficoll-PM70 ($\phi_{\mathrm{occ}}^{Ficoll} \approx 20\,\%$) mixture in $\mathbf{D_2O}$ as a function of diffusion-encoding gradient strength.**

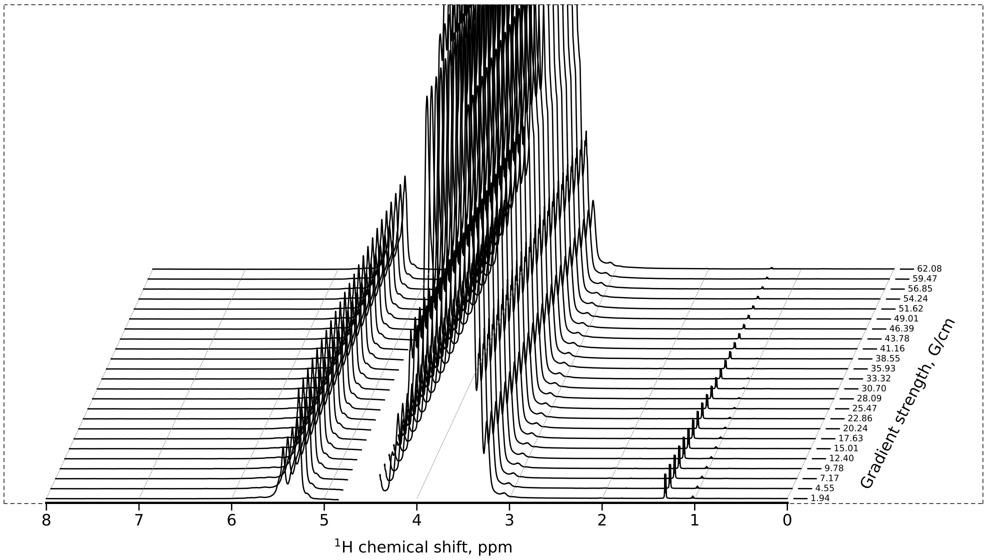

Figure S6: **Array of Dbppste ^{1}H NMR spectra of** 1.01 mM **alanine and Ficoll-PM70 ($\phi_{\text{occ}}^{Ficoll} \approx 30\,\%$) mixture in D_2O as a function of diffusion-encoding gradient strength.**

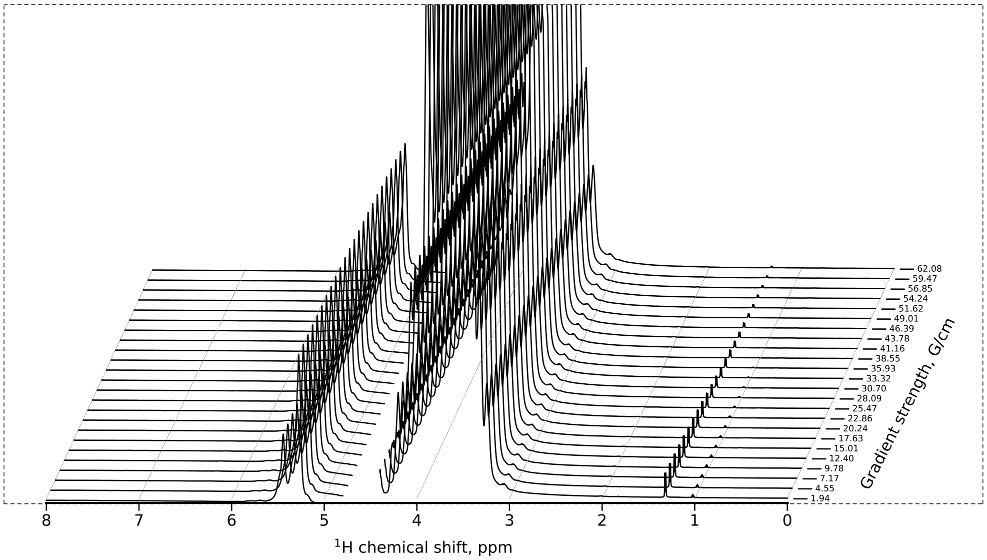

Figure S7: **Array of Dbppste ^{1}H NMR spectra of** 1.01 mM **alanine and Ficoll-PM70 ($\phi_{\text{occ}}^{Ficoll} \approx 40\,\%$) mixture in D_2O as a function of diffusion-encoding gradient strength.**

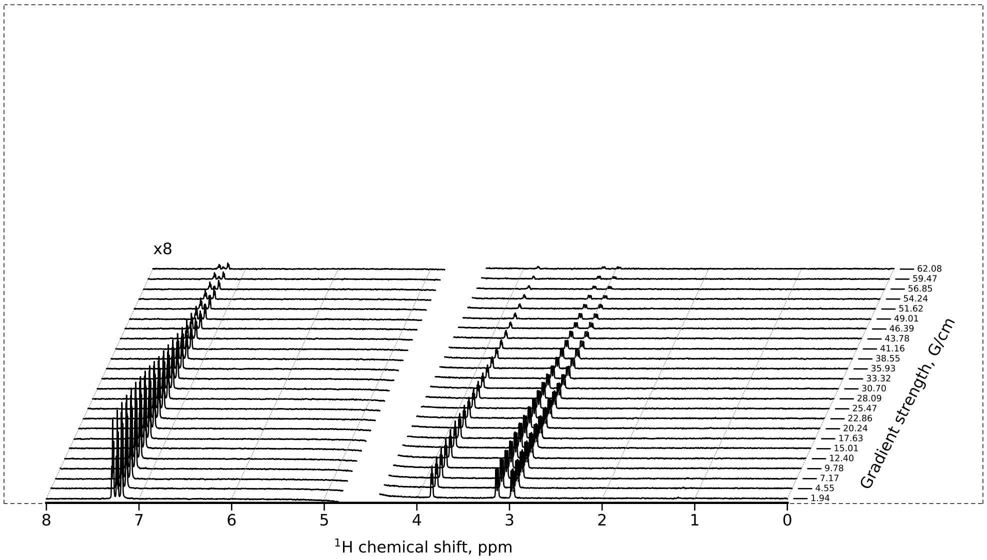

Figure S8: **Array of Dbppste ^{1}H NMR spectra of** $1.00\,\mathrm{mM}$ **phenylalanine solution in D_2O as a function of diffusion-encoding gradient strength.** The intensity has been upscaled by a factor of eight.

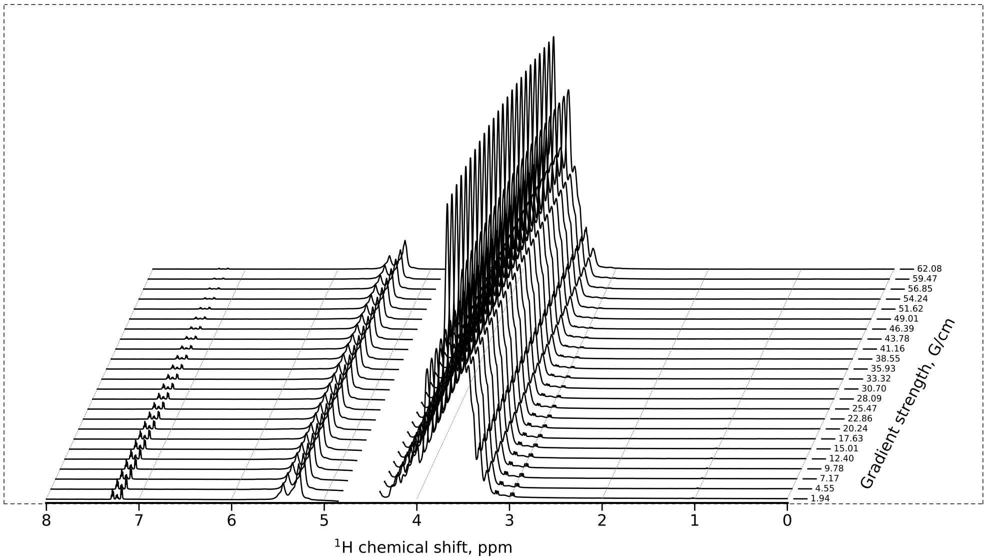

Figure S9: **Array of Dbppste ^{1}H NMR spectra of** $1.00\,\mathrm{mM}$ **phenylalanine and Ficoll-PM70 ($\phi_{\mathrm{occ}}^{Ficoll} \approx 10\,\%$) mixture in D_2O as a function of diffusion-encoding gradient strength.**

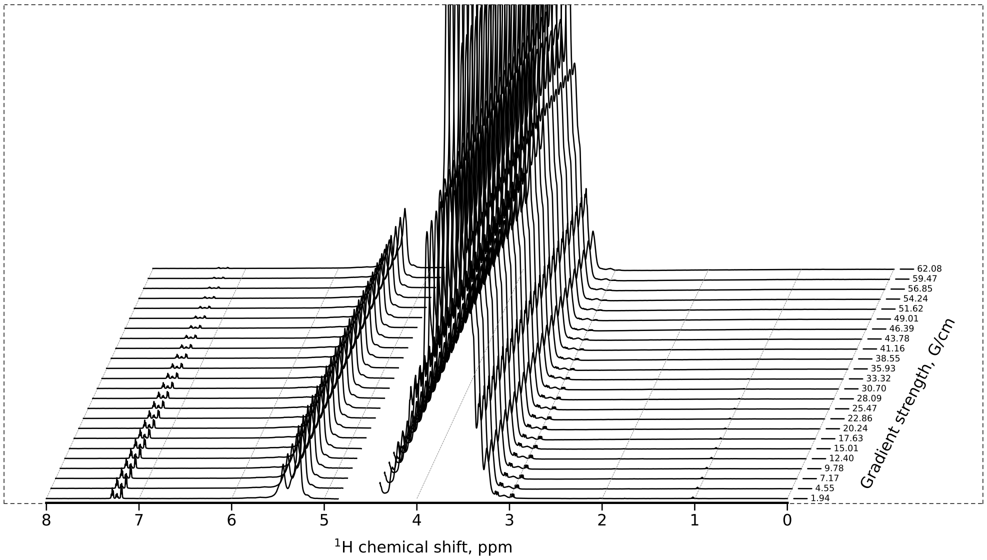

Figure S10: **Array of Dbppste ^{1}H NMR spectra of $1.00\,\text{mM}$ phenylalanine and Ficoll-PM70 ($\phi_{\text{occ}}^{Ficoll} \approx 20\,\%$) mixture in $\mathbf{D_2O}$ as a function of diffusion-encoding gradient strength.**

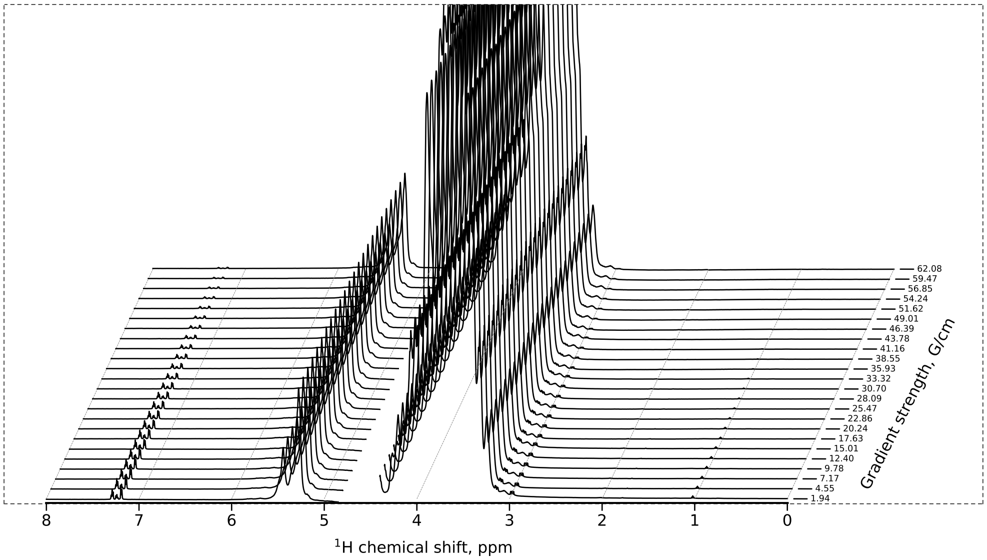

Figure S11: **Array of Dbppste ^{1}H NMR spectra of $1.00\,\text{mM}$ phenylalanine and Ficoll-PM70 ($\phi_{\text{occ}}^{Ficoll} \approx 30\,\%$) mixture in $\mathbf{D_2O}$ as a function of diffusion-encoding gradient strength.**

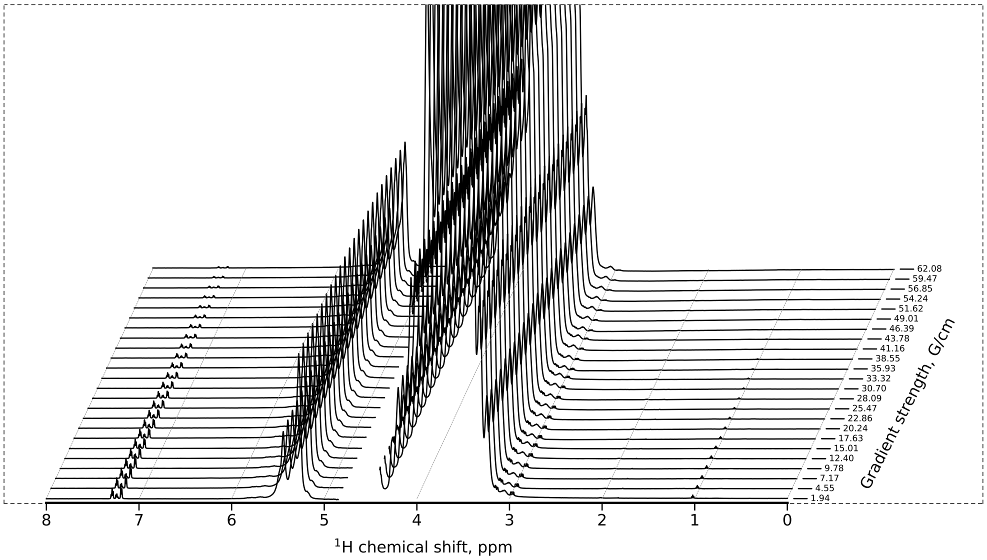

Figure S12: **Array of Dbppste ^{1}H NMR spectra of** 1.00 mM **phenylalanine and Ficoll-PM70 ($\phi_{\mathrm{occ}}^{Ficoll} \approx 40\,\%$) mixture in $\mathbf{D_2O}$ as a function of diffusion-encoding gradient strength.**

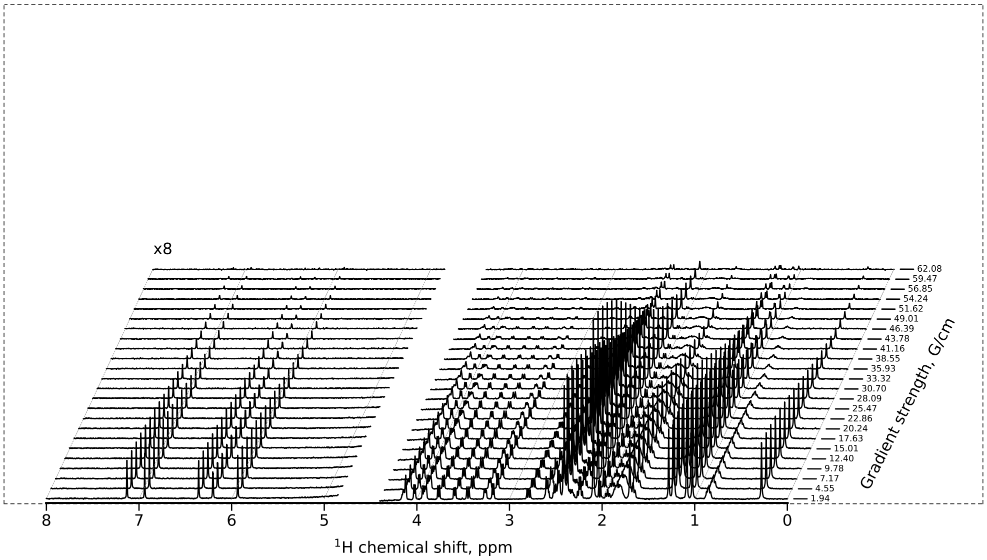

Figure S13: **Array of Dbppste ^{1}H NMR spectra of** 0.51 mM **cyanocobalamin solution in $\mathbf{D_2O}$ as a function of diffusion-encoding gradient strength.** The intensity has been upscaled by a factor of eight.

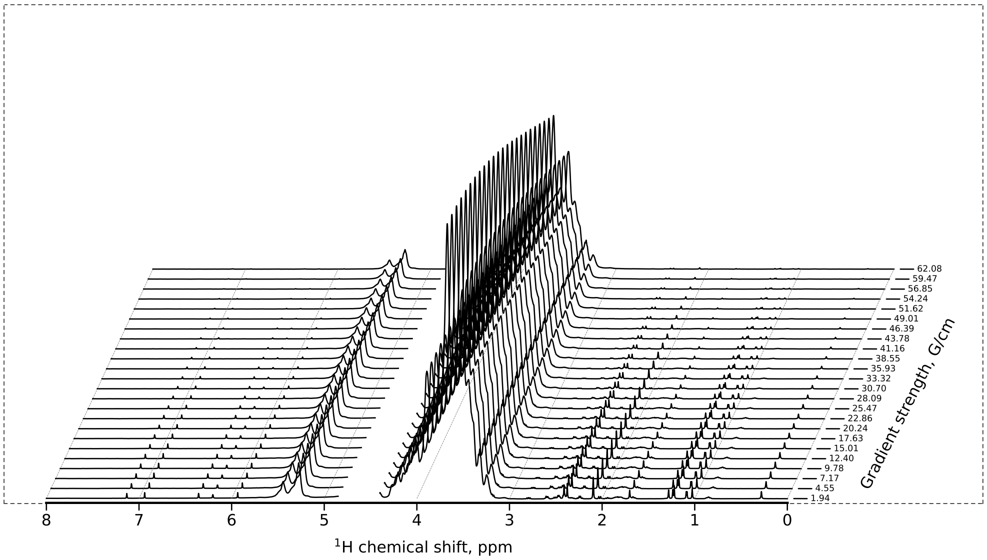

Figure S14: **Array of Dbppste ^{1}H NMR spectra of $0.51\,\mathrm{mM}$ cyanocobalamin and Ficoll-PM70 ($\phi_{\mathrm{occ}}^{Ficoll} \approx 10\,\%$) mixture in D_2O as a function of diffusion-encoding gradient strength.**

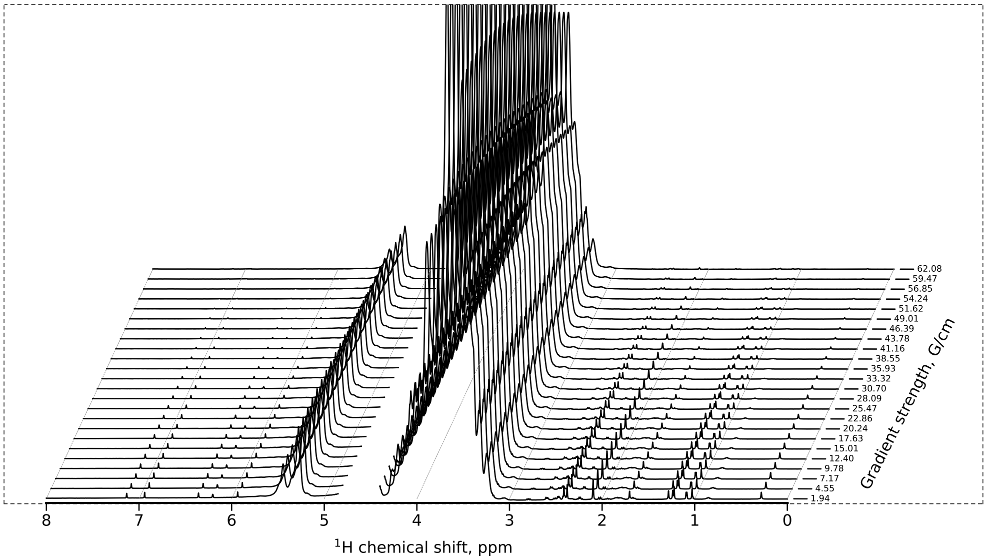

Figure S15: **Array of Dbppste ^{1}H NMR spectra of $0.51\,\mathrm{mM}$ cyanocobalamin and Ficoll-PM70 ($\phi_{\mathrm{occ}}^{Ficoll} \approx 20\,\%$) mixture in D_2O as a function of diffusion-encoding gradient strength.**

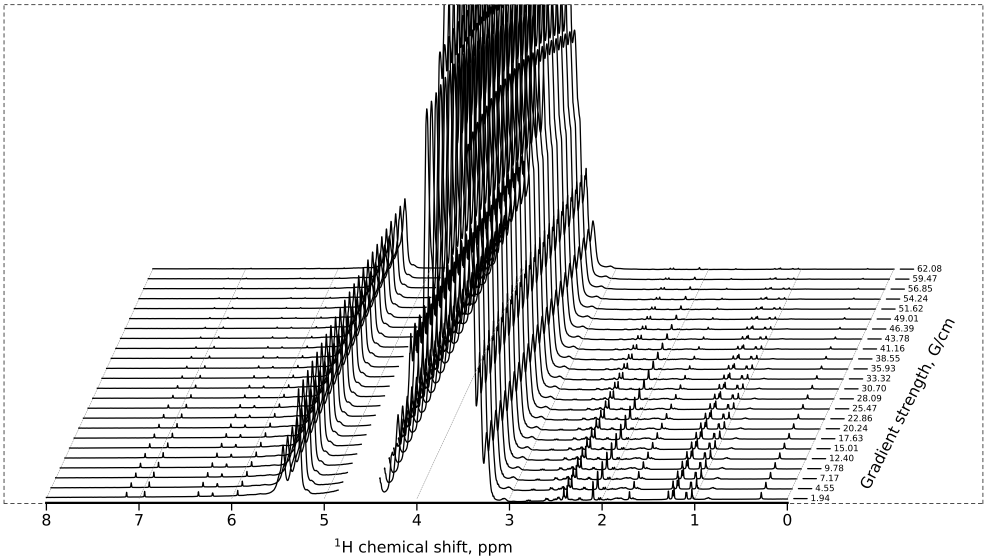

Figure S16: **Array of Dbppste ^{1}H NMR spectra of $0.51\,\mathrm{mM}$ cyanocobalamin and Ficoll-PM70 ($\phi_{\mathrm{occ}}^{Ficoll} \approx 30\,\%$) mixture in D_2O as a function of diffusion-encoding gradient strength.**

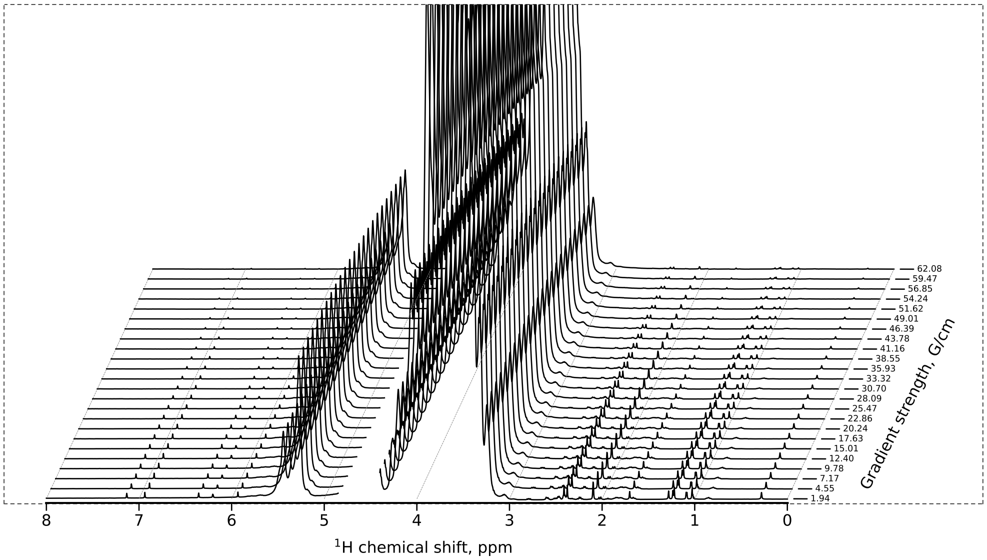

Figure S17: **Array of Dbppste ^{1}H NMR spectra of $0.51\,\mathrm{mM}$ cyanocobalamin and Ficoll-PM70 ($\phi_{\mathrm{occ}}^{Ficoll} \approx 40\,\%$) mixture in D_2O as a function of diffusion-encoding gradient strength.**

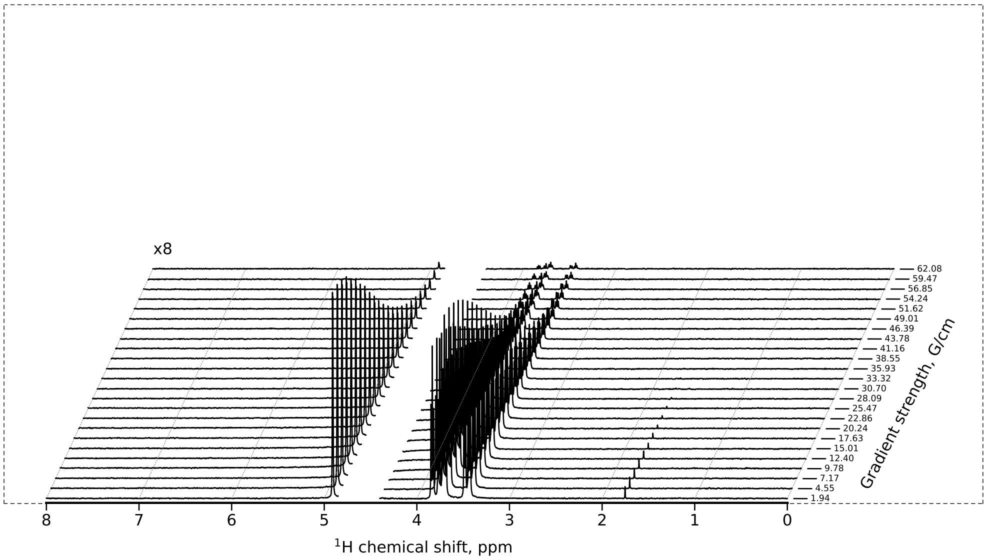

Figure S18: **Array of Dbppste ^{1}H NMR spectra of** $0.53\,\mathrm{mM}$ $\alpha-$**cyclodextrin solution in D_2O as a function of diffusion-encoding gradient strength.** The intensity has been upscaled by a factor of eight.

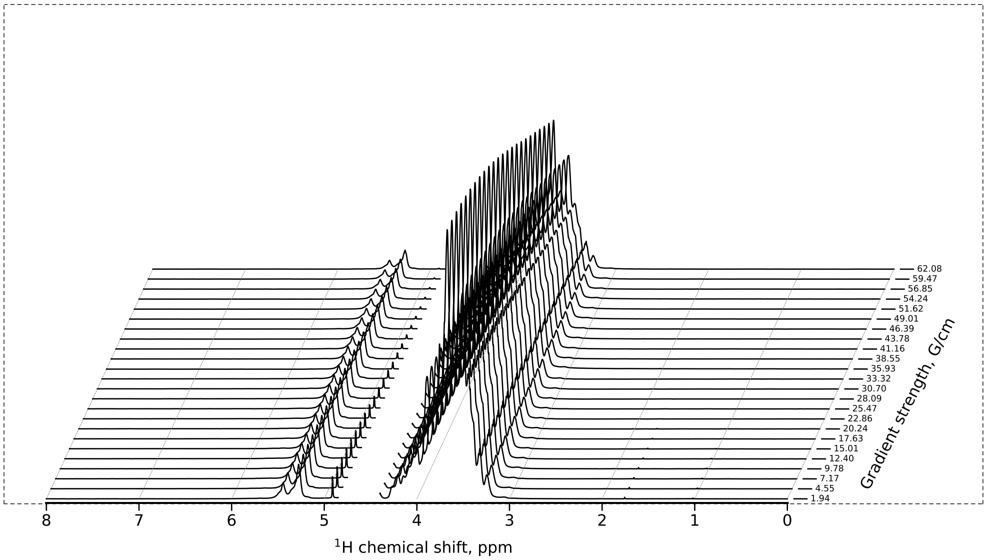

Figure S19: **Array of Dbppste ^{1}H NMR spectra of** $0.50\,\mathrm{mM}$ $\alpha-$**cyclodextrin and Ficoll-PM70 ($\phi_{\mathrm{occ}}^{Ficoll} \approx 10\,\%$) mixture in D_2O as a function of diffusion-encoding gradient strength.**

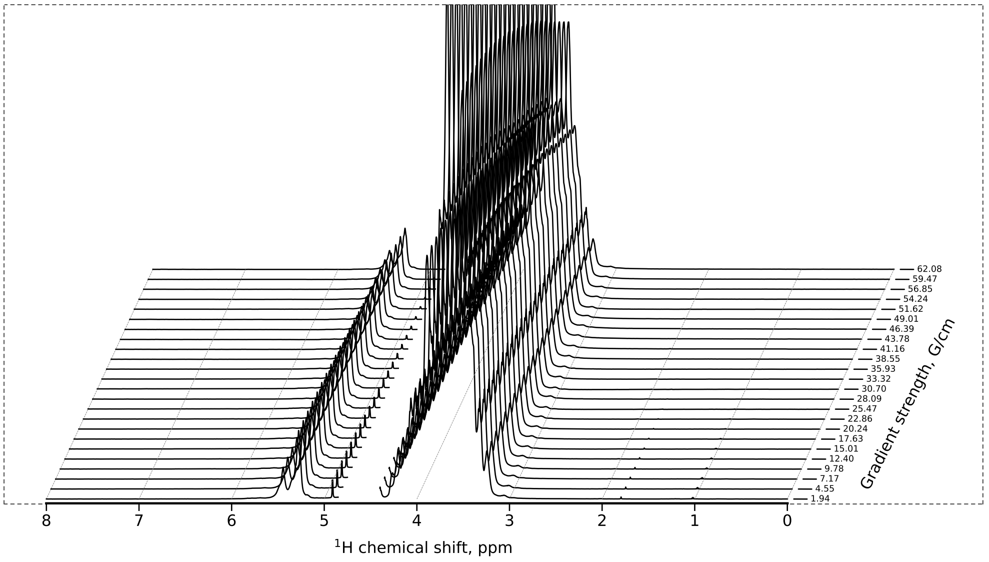

Figure S20: **Array of Dbppste ^{1}H NMR spectra of** $0.46\,\text{mM}$ $\alpha-$**cyclodextrin and Ficoll-PM70 (**$\phi_{\text{occ}}^{Ficoll} \approx 20\,\%$**) mixture in D_2O as a function of diffusion-encoding gradient strength.**

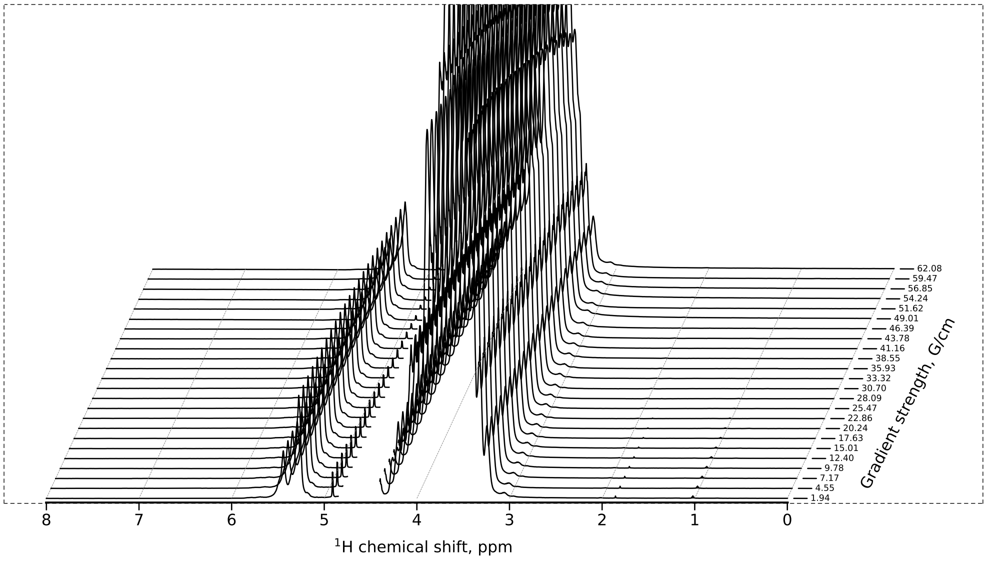

Figure S21: **Array of Dbppste ^{1}H NMR spectra of** $0.55\,\text{mM}$ $\alpha-$**cyclodextrin and Ficoll-PM70 (**$\phi_{\text{occ}}^{Ficoll} \approx 30\,\%$**) mixture in D_2O as a function of diffusion-encoding gradient strength.**

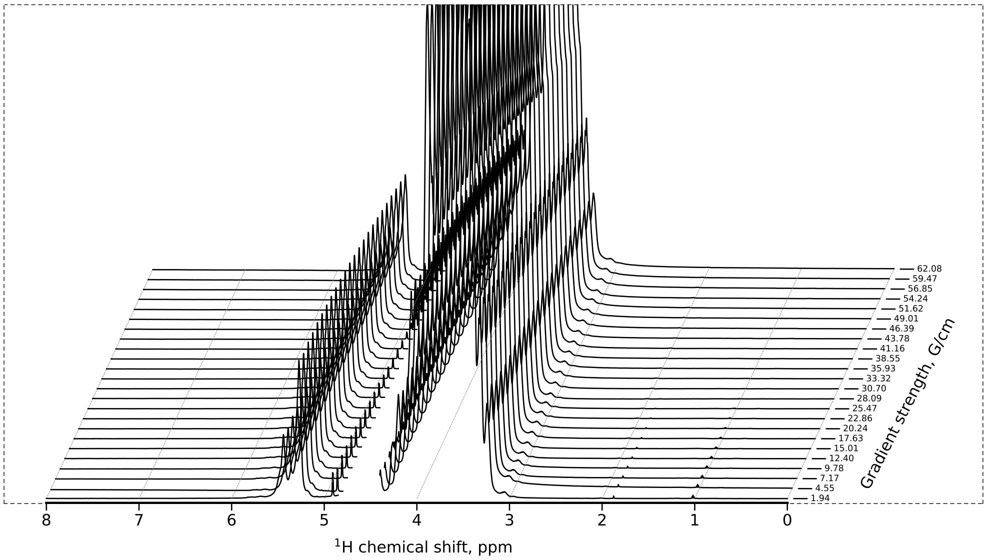

Figure S22: **Array of Dbppste ^{1}H NMR spectra of** $0.50\,\mathrm{mM}$ $\alpha-$**cyclodextrin and Ficoll-PM70 (**$\phi_{\mathrm{occ}}^{Ficoll} \approx 40\,\%$**) mixture in D_2O as a function of diffusion-encoding gradient strength.**

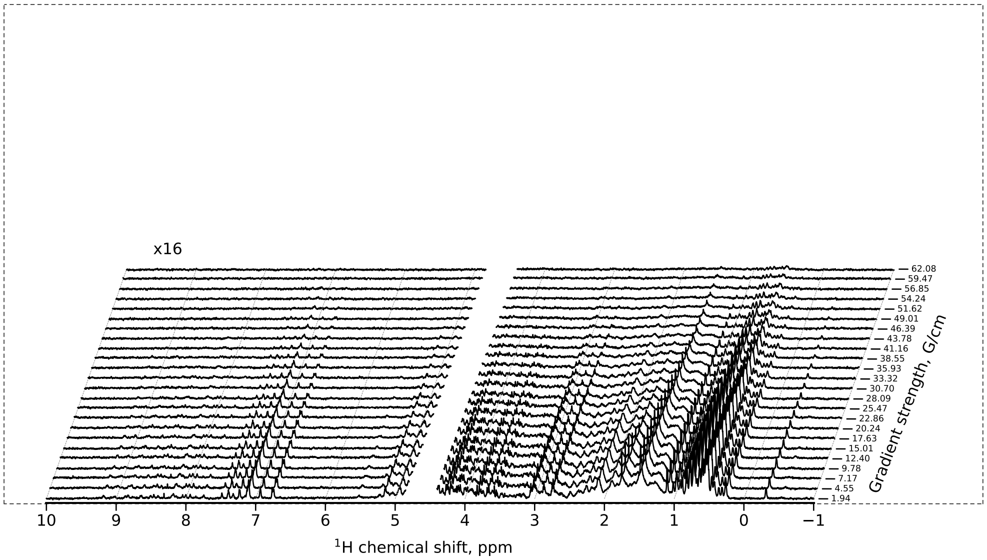

Figure S23: **Array of Dbppste ^{1}H NMR spectra of** $0.09\,\mathrm{mM}$ **ubiquitin (bovine) solution in D_2O as a function of diffusion-encoding gradient strength.** The intensity has been upscaled by a factor of sixteen.

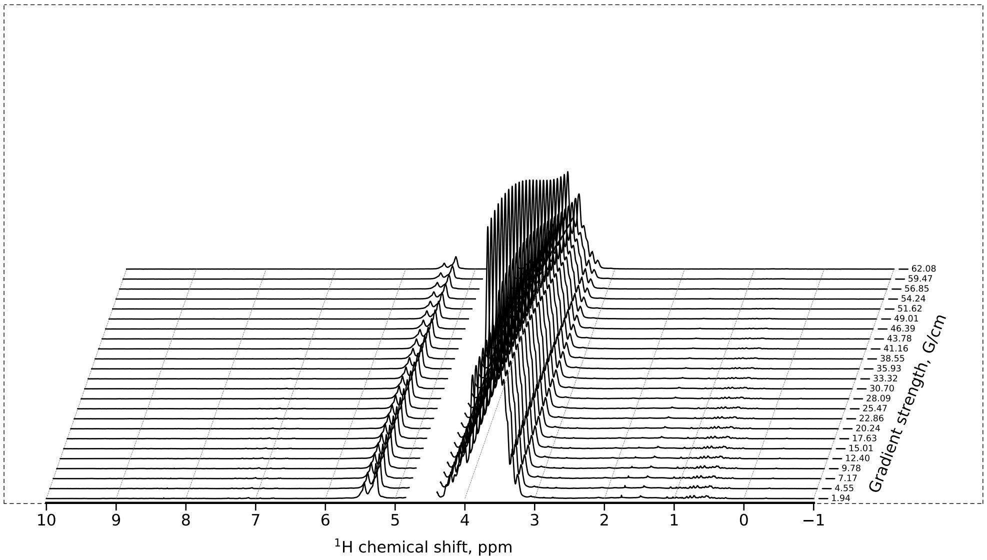

Figure S24: **Array of Dbppste ^{1}H NMR spectra of $0.10\,\mathrm{mM}$ ubiquitin (bovine) and Ficoll-PM70 ($\phi_{\mathrm{occ}}^{Ficoll} \approx 10\,\%$) mixture in $\mathbf{D_2O}$ as a function of diffusion-encoding gradient strength.**

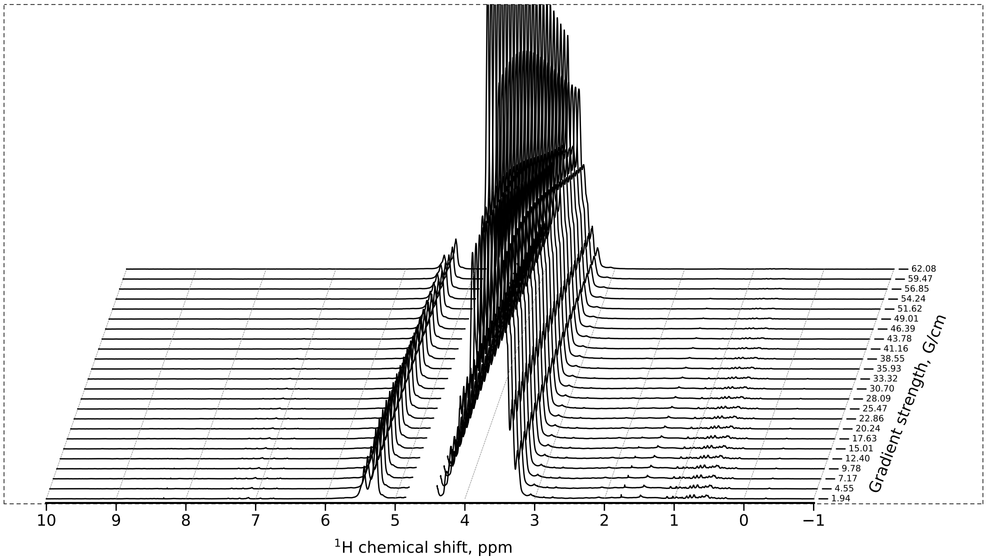

Figure S25: **Array of Dbppste ^{1}H NMR spectra of $0.09\,\mathrm{mM}$ ubiquitin (bovine) and Ficoll-PM70 ($\phi_{\mathrm{occ}}^{Ficoll} \approx 20\,\%$) mixture in $\mathbf{D_2O}$ as a function of diffusion-encoding gradient strength.**

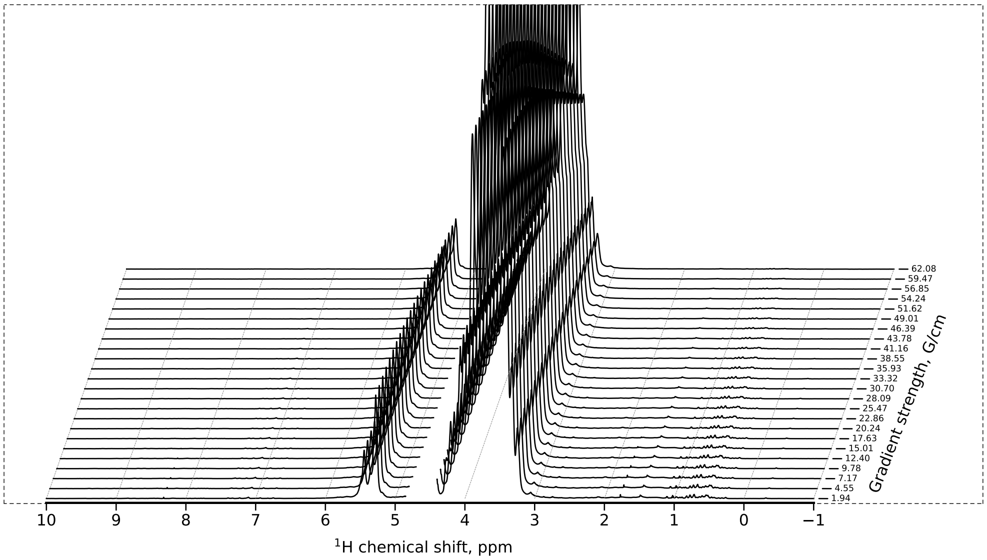

Figure S26: **Array of Dbppste ^{1}H NMR spectra of $0.08\,\mathrm{mM}$ ubiquitin (bovine) and Ficoll-PM70 ($\phi_{\mathrm{occ}}^{Ficoll} \approx 30\,\%$) mixture in D_2O as a function of diffusion-encoding gradient strength.**

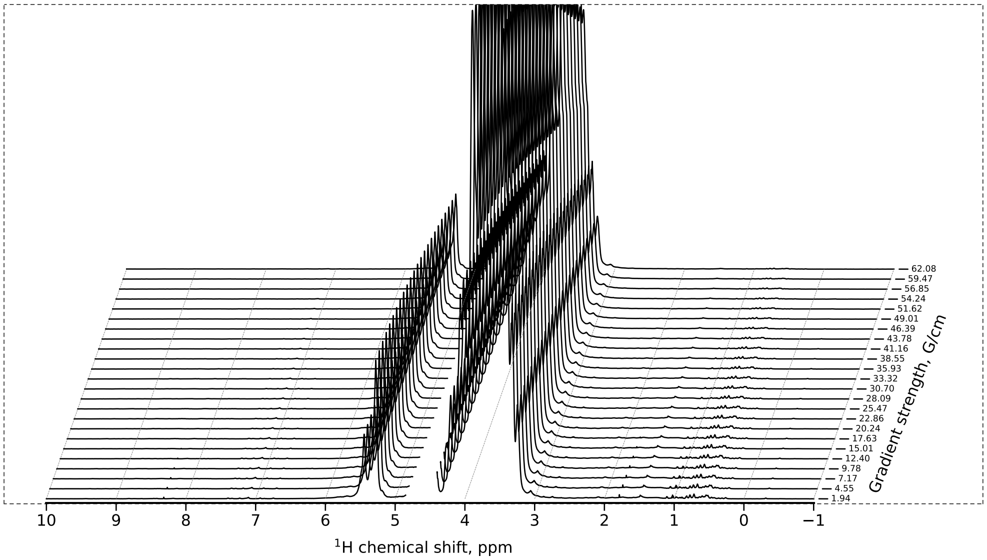

Figure S27: **Array of Dbppste ^{1}H NMR spectra of $0.08\,\mathrm{mM}$ ubiquitin (bovine) and Ficoll-PM70 ($\phi_{\mathrm{occ}}^{Ficoll} \approx 40\,\%$) mixture in D_2O as a function of diffusion-encoding gradient strength.**

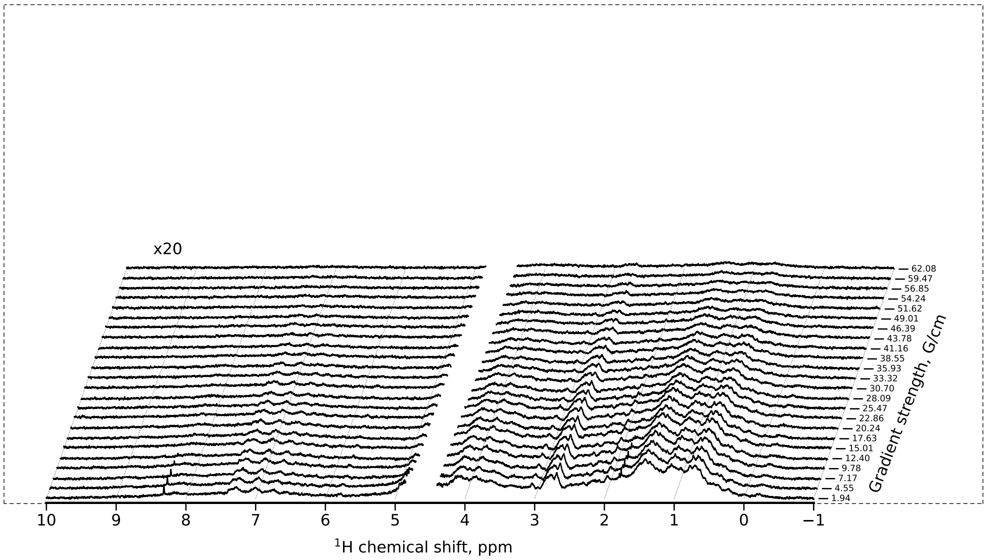

Figure S28: **Array of Dbppste ^{1}H NMR spectra of** $0.12\,\mathrm{mM}$ **Hemoglobin (bovine) solution in D_2O as a function of diffusion-encoding gradient strength.** The intensity has been upscaled by a factor of twenty.

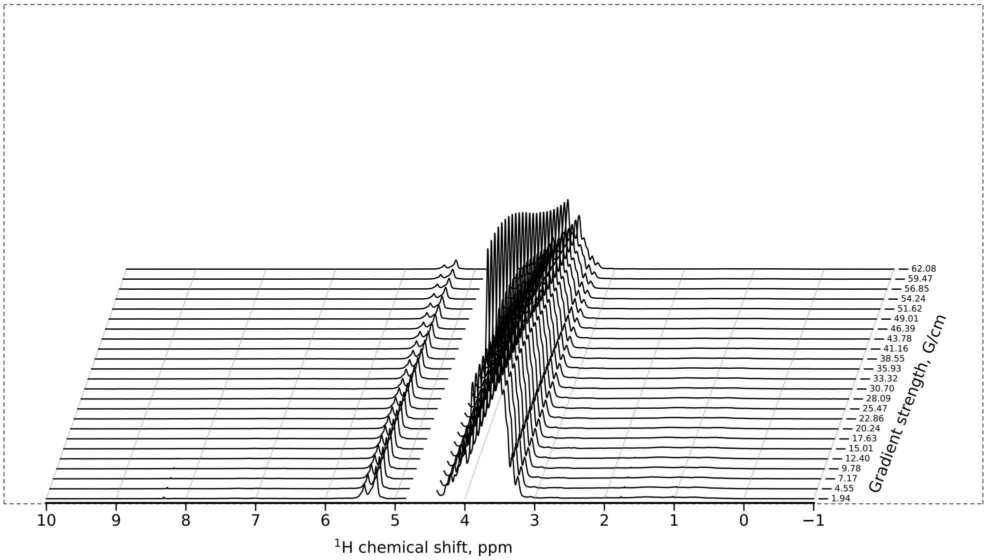

Figure S29: **Array of Dbppste ^{1}H NMR spectra of** $0.11\,\mathrm{mM}$ **Hemoglobin (bovine) and Ficoll-PM70 ($\phi_{\mathrm{occ}}^{Ficoll} \approx 10\,\%$) mixture in D_2O as a function of diffusion-encoding gradient strength.**

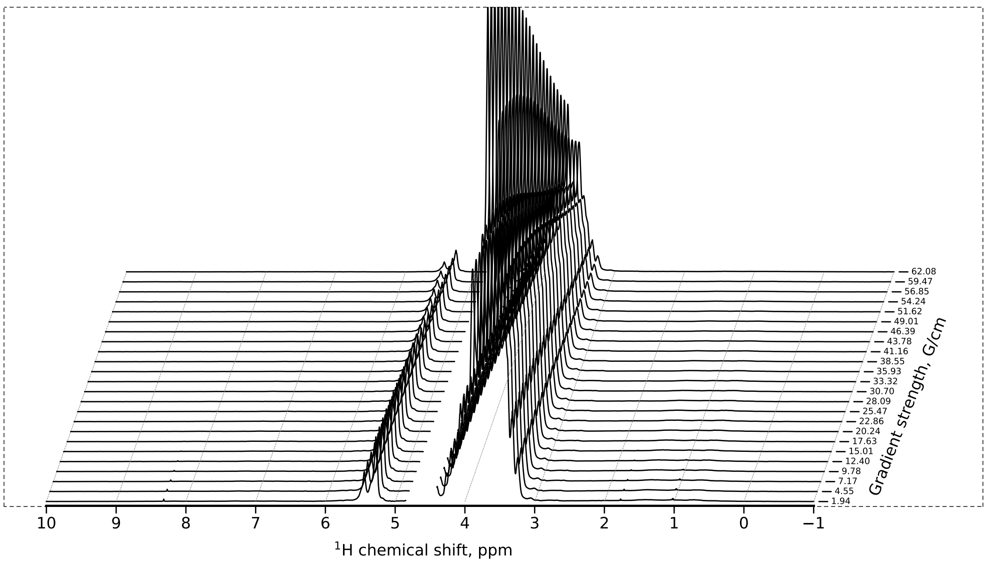

Figure S30: **Array of Dbppste ^{1}H NMR spectra of** $0.12\,\mathrm{mM}$ **Hemoglobin (bovine) and Ficoll-PM70 ($\phi_{\text{occ}}^{Ficoll} \approx 20\,\%$) mixture in $\mathbf{D_2O}$ as a function of diffusion-encoding gradient strength.**

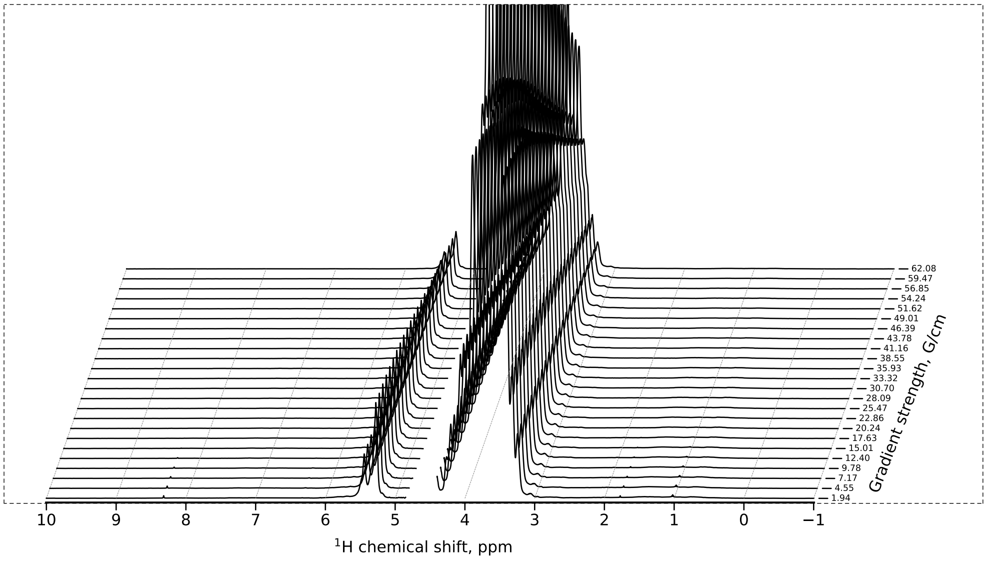

Figure S31: **Array of Dbppste ^{1}H NMR spectra of** $0.12\,\mathrm{mM}$ **Hemoglobin (bovine) and Ficoll-PM70 ($\phi_{\text{occ}}^{Ficoll} \approx 30\,\%$) mixture in $\mathbf{D_2O}$ as a function of diffusion-encoding gradient strength.**

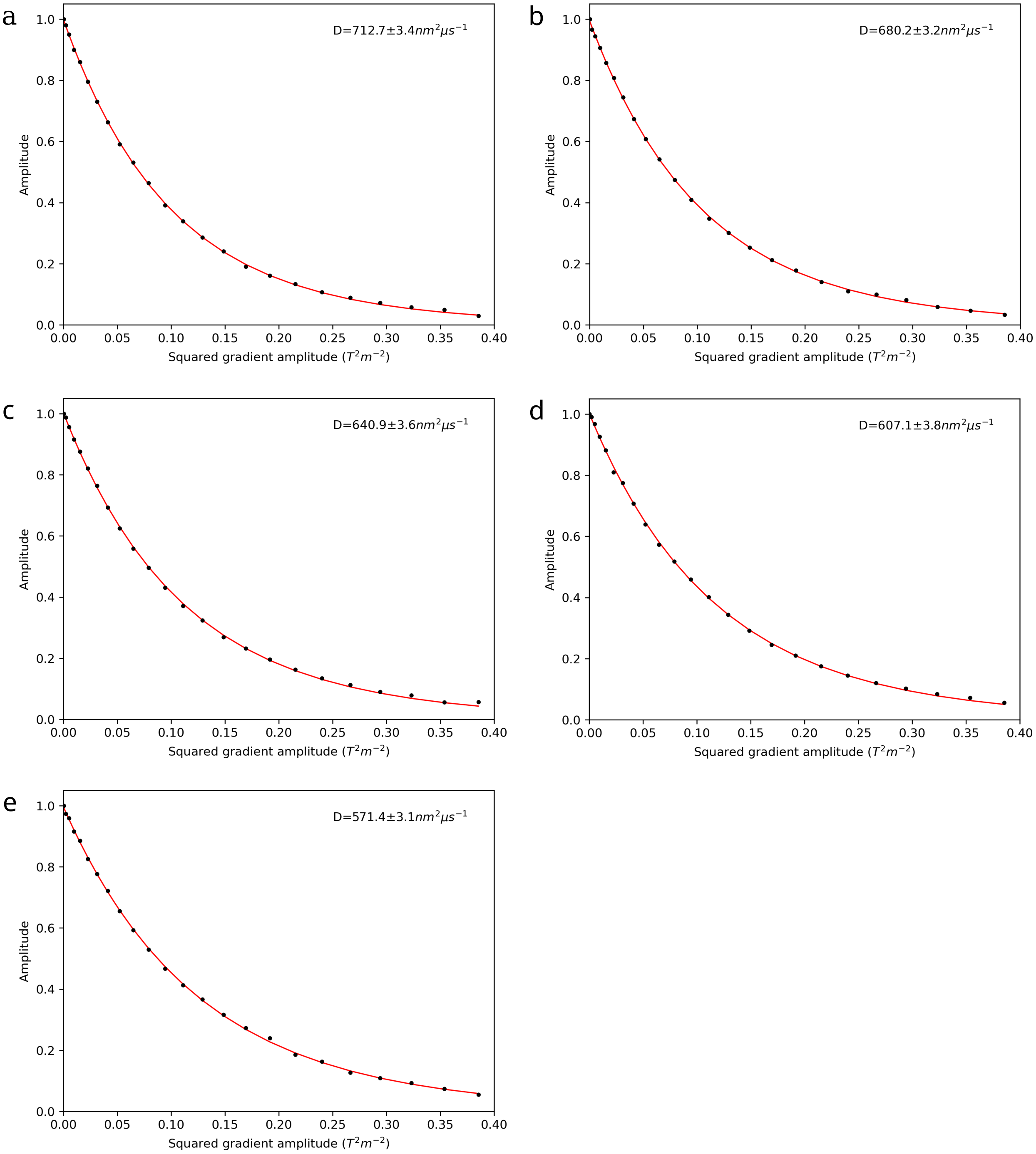

Figure S32: **Signal attenuation plots for alanine samples (a: $\phi_{\text{occ}}^{Ficoll} \approx 0\,\%$, b: $\phi_{\text{occ}}^{Ficoll} \approx 10\,\%$, c: $\phi_{\text{occ}}^{Ficoll} \approx 20\,\%$, d: $\phi_{\text{occ}}^{Ficoll} \approx 30\,\%$, e: $\phi_{\text{occ}}^{Ficoll} \approx 40\,\%$) as a function of the squared gradient amplitude**. The red curves show fit of the S1 equation. The calculated diffusion coefficients are displayed in the top right-hand corner of each plot.

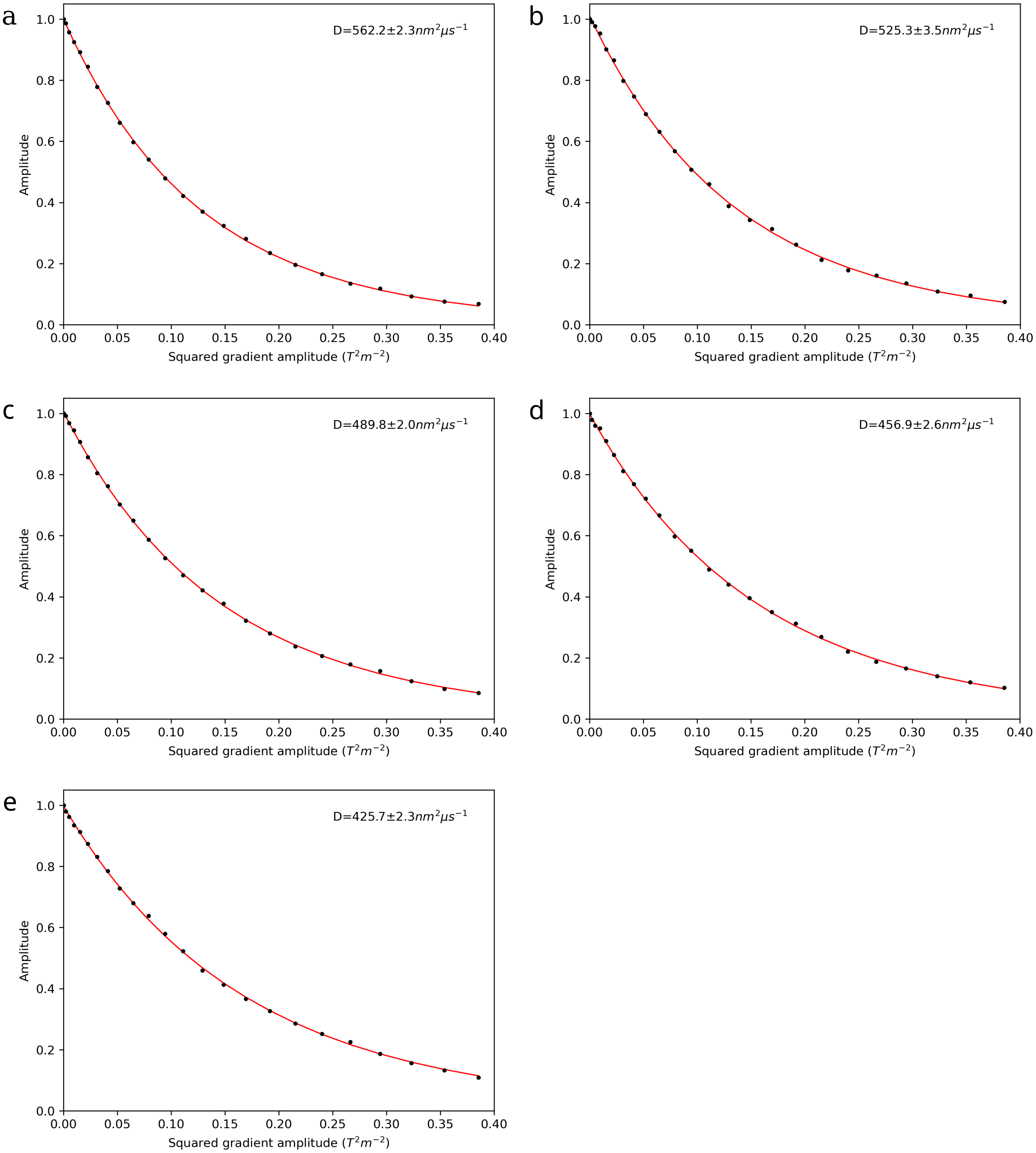

Figure S33: **Signal attenuation plots for phenylalanine samples (a: $\phi_{\mathrm{occ}}^{Ficoll} \approx 0\,\%$, b: $\phi_{\mathrm{occ}}^{Ficoll} \approx 10\,\%$, c: $\phi_{\mathrm{occ}}^{Ficoll} \approx 20\,\%$, d: $\phi_{\mathrm{occ}}^{Ficoll} \approx 30\,\%$, e: $\phi_{\mathrm{occ}}^{Ficoll} \approx 40\,\%$) as a function of the squared gradient amplitude**. The red curves show fit of the S1 equation. The calculated diffusion coefficients are displayed in the top right-hand corner of each plot.

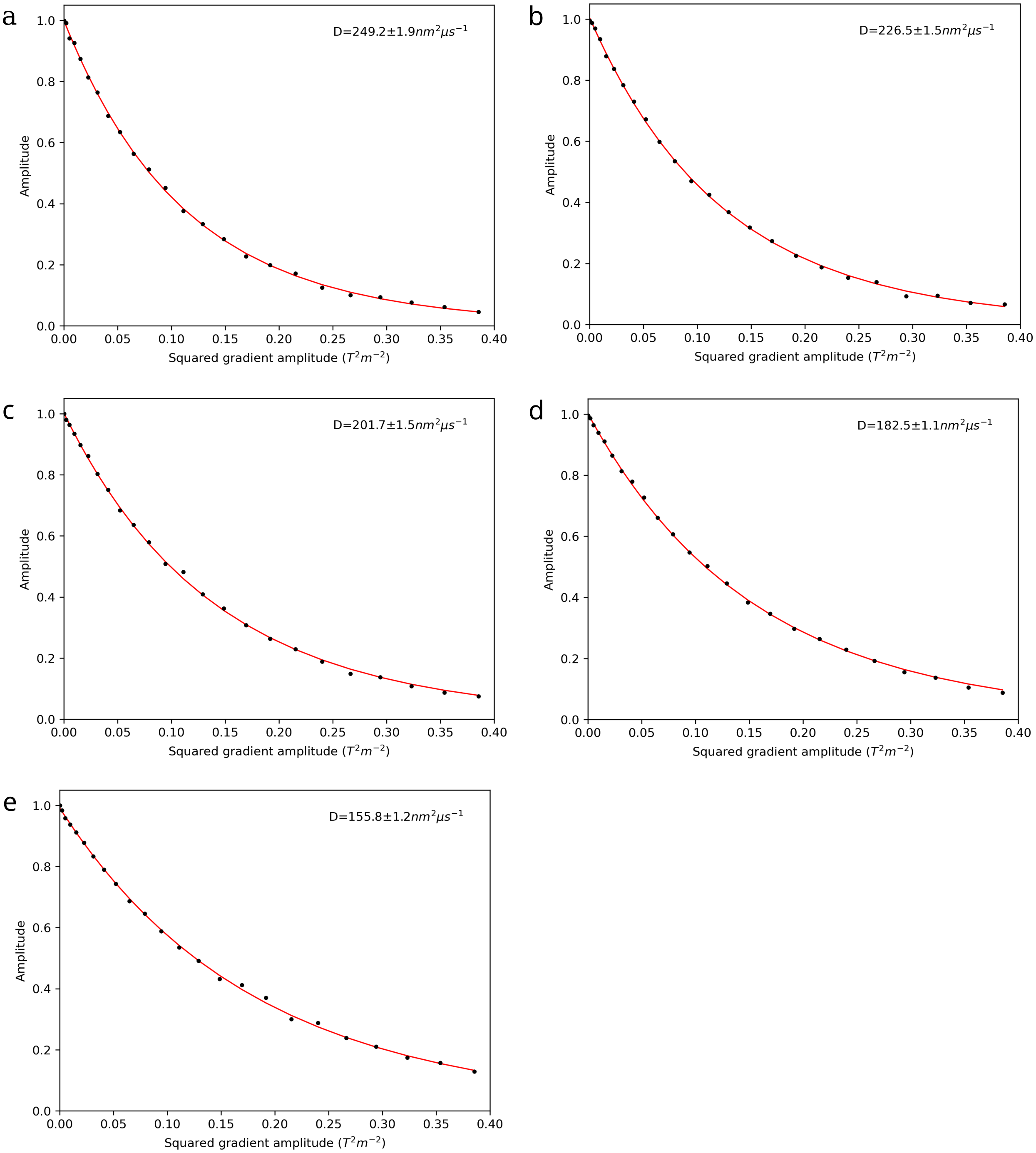

Figure S34: **Signal attenuation plots for cyanocobalamin samples (a: $\phi_{\text{occ}}^{Ficoll} \approx 0\,\%$, b: $\phi_{\text{occ}}^{Ficoll} \approx 10\,\%$, c: $\phi_{\text{occ}}^{Ficoll} \approx 20\,\%$, d: $\phi_{\text{occ}}^{Ficoll} \approx 30\,\%$, e: $\phi_{\text{occ}}^{Ficoll} \approx 40\,\%$) as a function of the squared gradient amplitude**. The red curves show fit of the S1 equation. The calculated diffusion coefficients are displayed in the top right-hand corner of each plot.

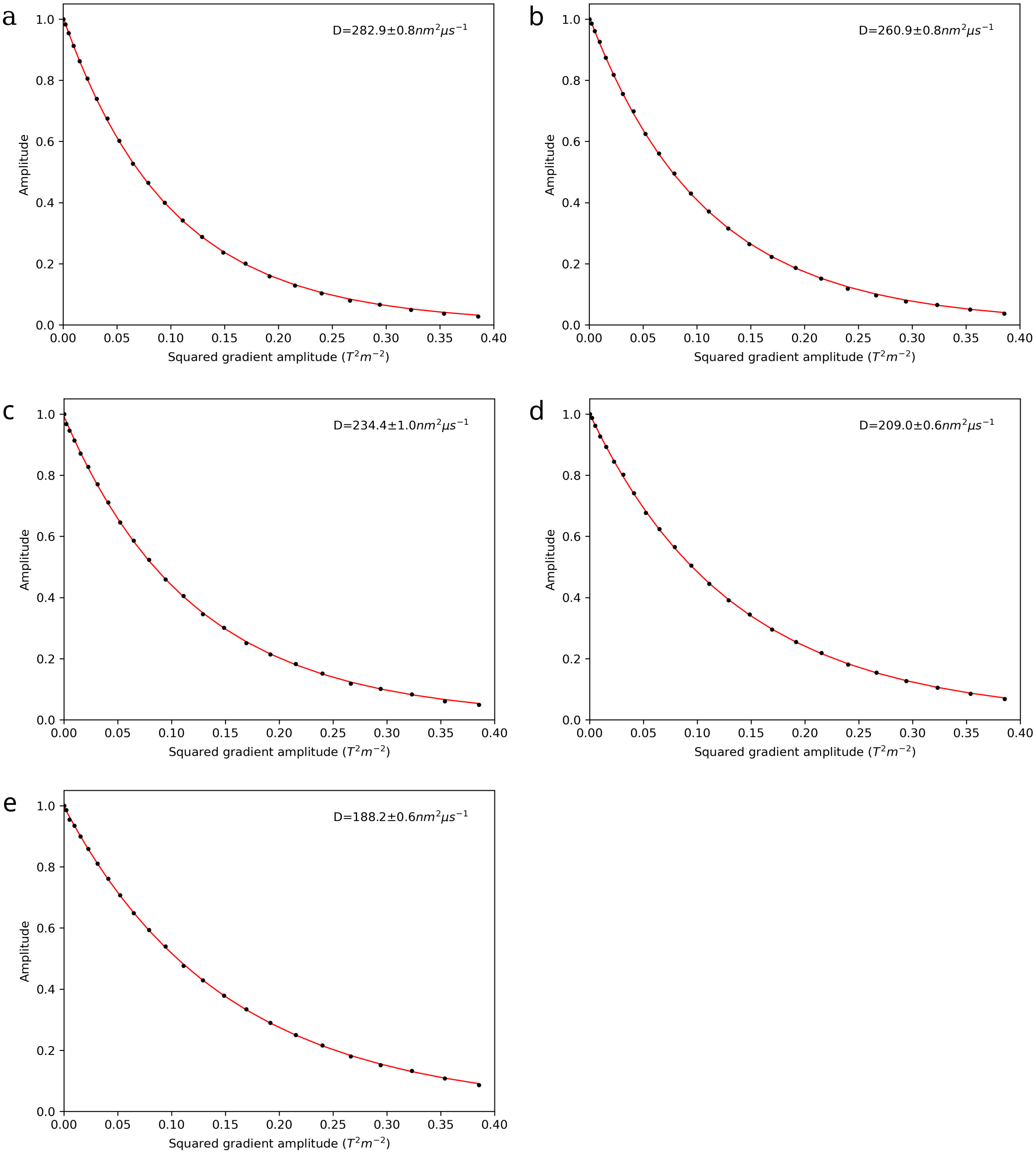

Figure S35: **Signal attenuation plots for α-cyclodextrin samples (a: $\phi_{\text{occ}}^{Ficoll} \approx 0\,\%$, b: $\phi_{\text{occ}}^{Ficoll} \approx 10\,\%$, c: $\phi_{\text{occ}}^{Ficoll} \approx 20\,\%$, d: $\phi_{\text{occ}}^{Ficoll} \approx 30\,\%$, e: $\phi_{\text{occ}}^{Ficoll} \approx 40\,\%$) as a function of the squared gradient amplitude**. The red curves show fit of the S1 equation. The calculated diffusion coefficients are displayed in the top right-hand corner of each plot.

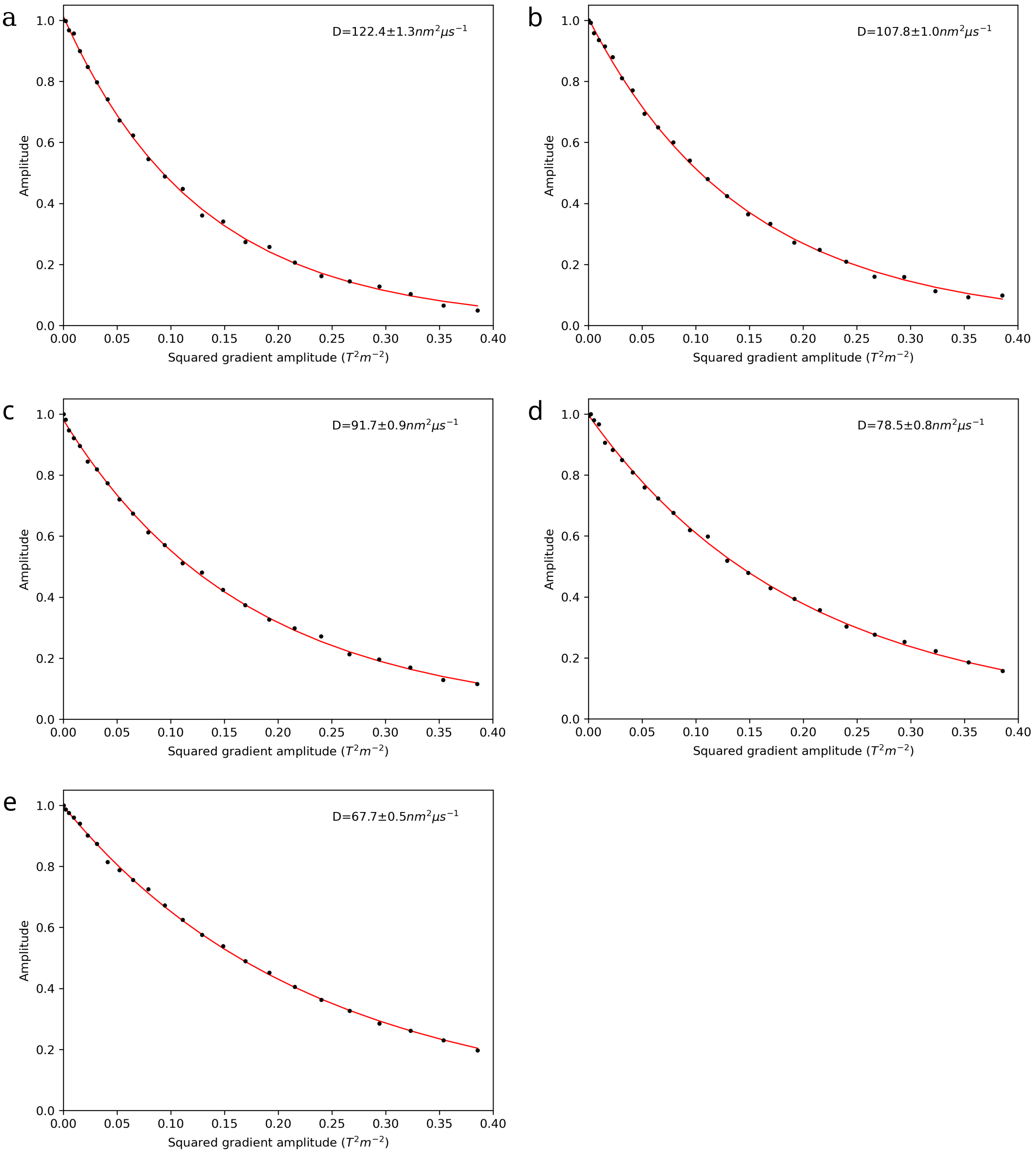

Figure S36: **Signal attenuation plots for ubiquitin samples (a: $\phi_{\text{occ}}^{Ficoll} \approx 0\,\%$, b: $\phi_{\text{occ}}^{Ficoll} \approx 10\,\%$, c: $\phi_{\text{occ}}^{Ficoll} \approx 20\,\%$, d: $\phi_{\text{occ}}^{Ficoll} \approx 30\,\%$, e: $\phi_{\text{occ}}^{Ficoll} \approx 40\,\%$) as a function of the squared gradient amplitude**. The red curves show fit of the S1 equation. The calculated diffusion coefficients are displayed in the top right-hand corner of each plot.

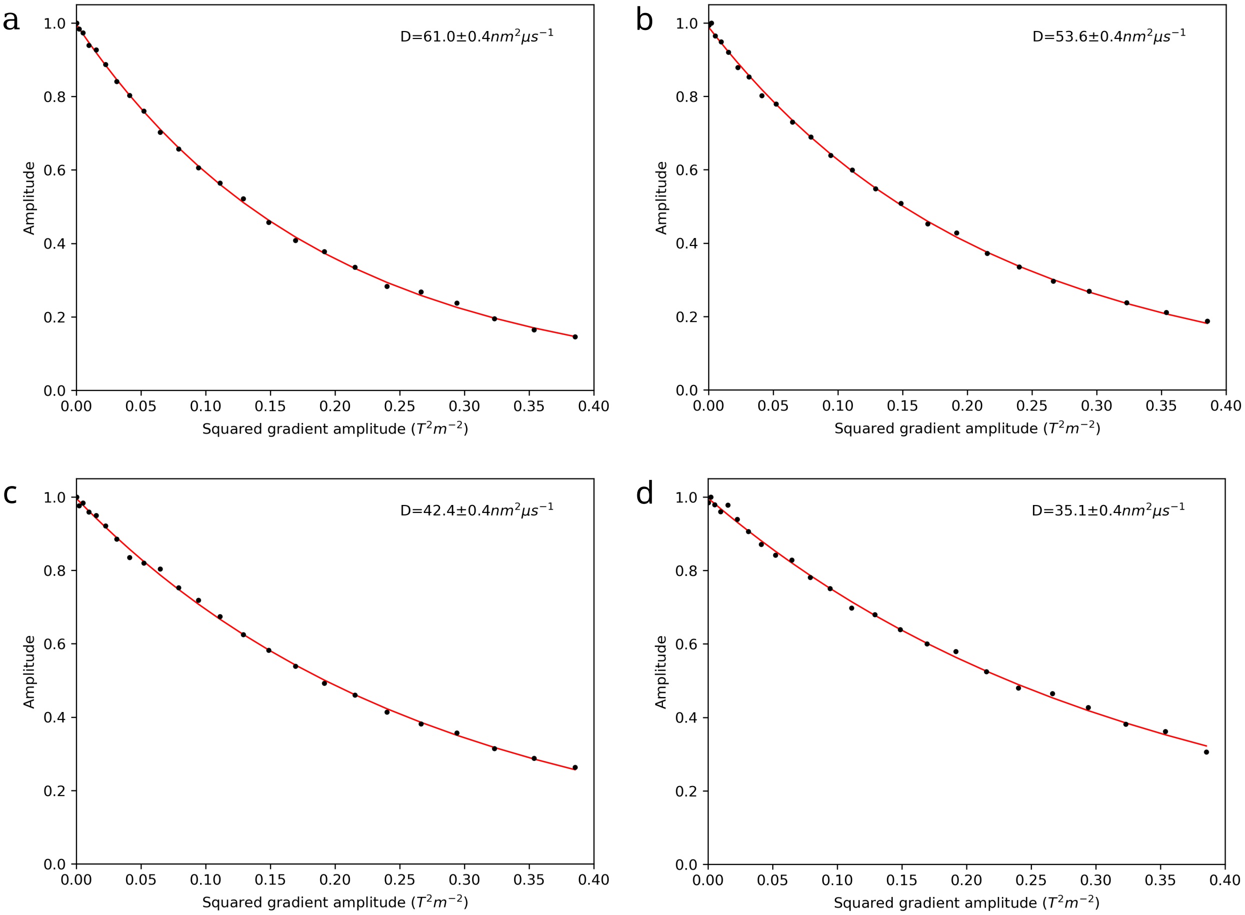

Figure S37: **Signal attenuation plots for hemoglobin samples (a: $\phi_{\mathrm{occ}}^{Ficoll} \approx 0\,\%$, b: $\phi_{\mathrm{occ}}^{Ficoll} \approx 10\,\%$, c: $\phi_{\mathrm{occ}}^{Ficoll} \approx 20\,\%$, d: $\phi_{\mathrm{occ}}^{Ficoll} \approx 30\,\%$) as a function of the squared gradient amplitude**. The red curves show fit of the S1 equation. The calculated diffusion coefficients are displayed in the top right-hand corner of each plot.

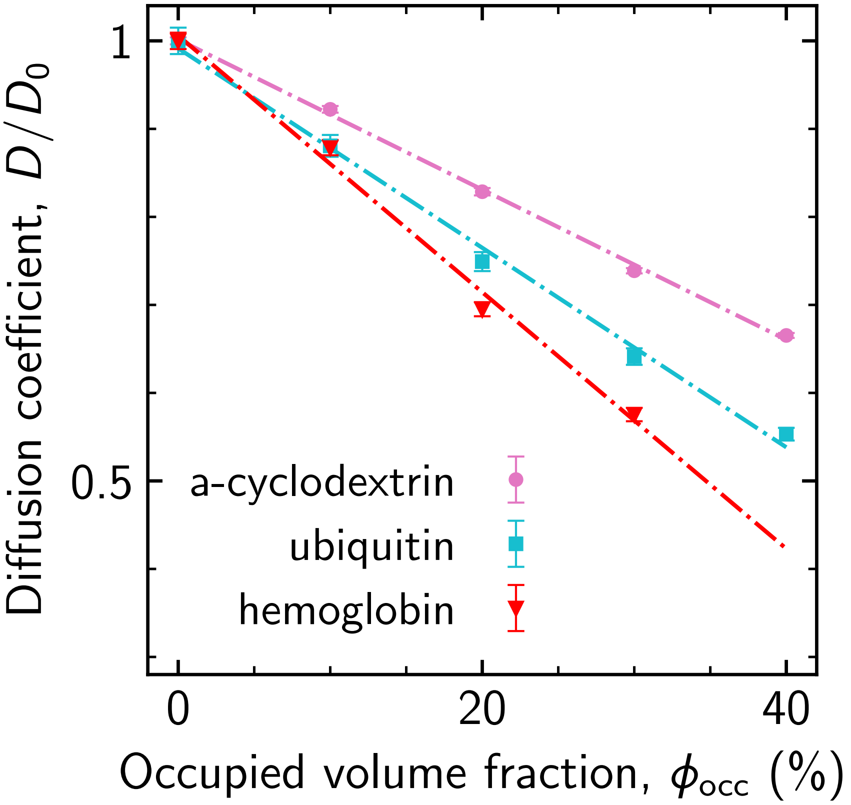

Figure S38: **NMR results for tracer diffusivity under crowding.** Diffusion coefficients of a-cyclodextrin (hydrodynamic radius $R_H \approx 0.70$ nm), ubiquitin ($R_H \approx 1.63$ nm), and hemoglobin ($R_H \approx 3.27$ nm) as functions of the occupied volume fraction ϕ_{occ} of Ficoll70 (see Table S1 for details). D_0 is the diffusion coefficient for $\phi_{occ} = 0$. The lines show the results of fitting Eq. (2) (main text) to the NMR data.

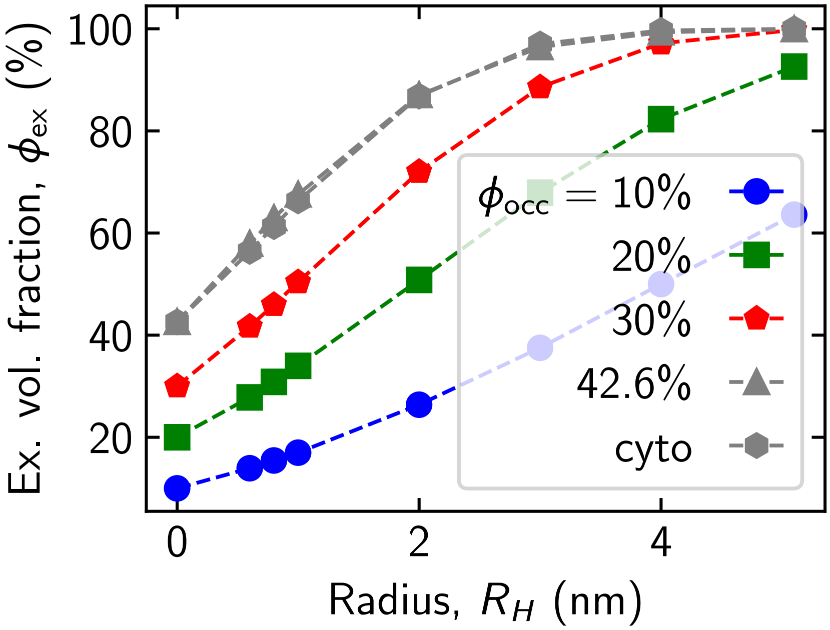

Figure S39: **Excluded volumes** *vs.* **tracer radius.** Excluded volume fraction as a function of the tracer radius obtained by Monte Carlo (MC) simulations for crowding with Ficoll-70 (hydrodynamic radius $R_{\rm H} = 5.1\,\rm nm$) and for the cytoplasm model of Ando and Skolnick.[23]

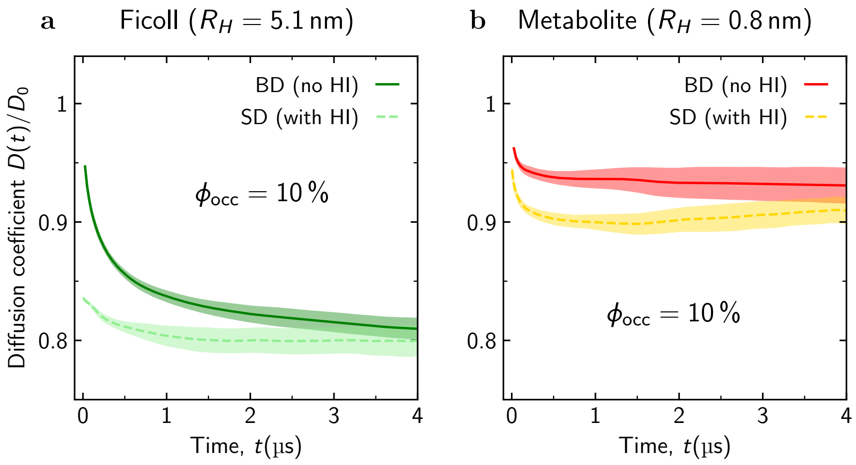

Figure S40: **Effect of hydrodynamic interactions.** Time-dependent diffusion coefficient $D(t)/D_0$ of Ficoll (**a**) and metabolites (**b**) in a mixture of metabolites and Ficoll simulated with and without hydrodynamic interactions. The system with hydrodynamic interactions was simulated using the F-version Stokesian dynamics. The occupied volume fraction $\phi_{occ} \approx 10\,\%$.

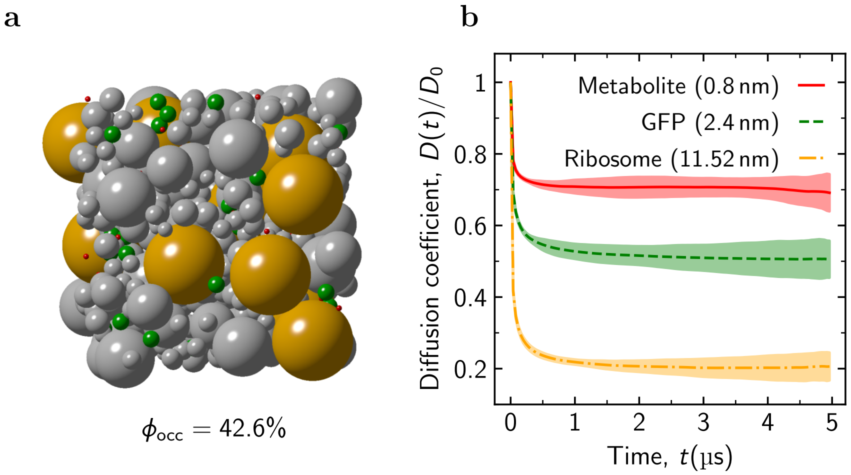

Figure S41: **Diffusion in the *E. coli* cytoplasm.** (**a**) Snapshot from BD simulations of metabolites in the *E. coli* cytoplasm model of ref. 23,28. The red, green and orange spheres represent metabolites, GFP and ribosomes, respectively, and the remaining macromolecules are shown in grey. (**b**) Time-dependent diffusion coefficient of metabolites ($R_H = 0.8$ nm), GFP ($R_H = 2.4$ nm), and ribosomes ($R_H = 11.52$ nm) expressed in terms of the corresponding diffusion coefficient D_0 in infinite dilution. The color code is the same as in panel (a).

References

(1) Millero, F. J.; Dexter, Roger.; Hoff, Edward. Density and Viscosity of Deuterium Oxide Solutions from 5-70.Deg. *J. Chem. Eng. Data* **1971**, *16*, 85–87.

(2) Connell, M. A.; Bowyer, P. J.; Adam Bone, P.; Davis, A. L.; Swanson, A. G.; Nilsson, M.; Morris, G. A. Improving the Accuracy of Pulsed Field Gradient NMR Diffusion Experiments: Correction for Gradient Non-Uniformity. *J. Magn. Reson.* **2009**, *198*, 121–131.

(3) Mills, R. Self-Diffusion in Normal and Heavy Water in the Range 1-45.Deg. *J. Phys. Chem.* **1973**, *77*, 685–688.

(4) Helmus, J. J.; Jaroniec, C. P. Nmrglue: An Open Source Python Package for the Analysis of Multidimensional NMR Data. *J Biomol NMR* **2013**, *55*, 355–367.

(5) Nilsson, M. The DOSY Toolbox: A New Tool for Processing PFG NMR Diffusion Data. *J. Magn. Reson.* **2009**, *200*, 296–302.

(6) Virtanen, P. *et al.* SciPy 1.0: Fundamental Algorithms for Scientific Computing in Python. *Nat. Methods* **2020**, *17*, 261–272.

(7) Ermak, D. L.; McCammon, J. A. Brownian dynamics with hydrodynamic interactions. *J. Chem. Phys.* **1978**, *69*, 1352–1360.

(8) Długosz, M.; Trylska, J. Diffusion in crowded biological environments: Applications of Brownian dynamics. *BMC Biophys.* **2011**, *4*, 3.

(9) Huber, G. A.; McCammon, J. A. Brownian Dynamics Simulations of Biological Molecules. *Trends Chem.* **2019**, *1*, 727–738.

(10) Skóra, T.; Słyk, E.; Kondrat, S. pyBrown: A Versatile Brownian and Stokesian Dynamics Package for Simulations of Diffusion and Reactions. *To be published (https://github.com/tskora/pyBrown).*

(11) Skóra, T.; Vaghefikia, F.; Fitter, J.; Kondrat, S. Macromolecular Crowding: How Shape and Interactions Affect Diffusion. *J. Phys. Chem. B* **2020**, *124*, 7537–7543.

(12) Skóra, T.; N. Popescu, M.; Kondrat, S. Conformation-Changing Enzymes and Macromolecular Crowding. *Phys. Chem. Chem. Phys.* **2021**, *23*, 9065–9069.

(13) Słyk, E.; Skóra, T.; Kondrat, S. How Macromolecules Softness Affects Diffusion under Crowding. *Soft Matter* **2022**, *18*, 5366–5370.

(14) Słyk, E.; Skóra, T.; Kondrat, S. Minimal Coarse-Grained Model for Immunoglobulin G: Diffusion and Binding under Crowding. *J. Phys. Chem. B* **2023**, *127*, 7442–7448.

(15) Durlofsky, L.; Brady, J. F.; Bossis, G. Dynamic simulation of hydrodynamically interacting Dynamic simulation of hydrodynamically interacting particles. *J. Fluid Mech.* **1987**, 21–49.

(16) Phillips, R. J.; Brady, J. F.; Bossis, G. Hydrodynamic transport properties of hard-sphere dispersions. I. Suspensions of freely mobile particles. *Phys. Fluids* **1988**, *31*, 3462.

(17) Brady, J. F.; Phillips, R. J.; Bossis, G. Dynamic simulation of hydrodynamically interacting suspensions. *J. Fluid Mech.* **1988**, *195*, 257–280.

(18) Brady, J. F.; Bossis, G. Stokesian Dynamics. *Annu. Rev. Fluid Mech.* **1988**, *20*, 111–157.

(19) Rotne, J.; Prager, S. Variational Treatment of Hydrodynamic Interaction in Polymers. *J. Chem. Phys.* **1969**, *50*, 4831–4837.

(20) Yamakawa, H. Transport properties of polymer chains in dilute solution: hydrodynamic interaction. *J. Chem. Phys.* **1970**, *53*, 436–443.

(21) Zuk, P. J.; Wajnryb, E.; Mizerski, K. A.; Szymczak, P. Rotne-Prager-Yamakawa approximation for different-sized particles in application to macromolecular bead models. *J. Fluid Mech.* **2014**, *741*, R5.

(22) Banchio, A. J.; Brady, J. F. Accelerated Stokesian dynamics: Brownian motion. *J. Chem. Phys.* **2003**, *118*, 10323–10332.

(23) Ando, T.; Skolnick, J. Crowding and Hydrodynamic Interactions Likely Dominate in Vivo Macromolecular Motion. *Proc Natl Acad Sci U S A* **2010**, *107*, 18457–18462.

(24) Smith, E. R.; Snook, I. K.; Van Megen, W. Hydrodynamic interactions in Brownian dynamics. *Phys. A Stat. Mech. its Appl.* **1987**, *143*, 441–467.

(25) Jeffrey, D. J.; Onishi, Y. Calculation of the resistance and mobility functions for two unequal rigid spheres in low-Reynolds-number flow. *J. Fluid Mech.* **1984**, *139*, 261–290.

(26) Cichocki, B.; Ekiel-Jeżewska, M. L.; Wajnryb, E. Lubrication corrections for three-particle contribution to short-time self-diffusion coefficients in colloidal dispersions. *J. Chem. Phys.* **1999**, *111*, 3265–3273.

(27) Skóra, T. Diffusion and Reactions under Crowding: Theory and Simulations. Ph.D. thesis, Institute of Physical Chemistry, Warsaw, 2023.

(28) Ridgway, D.; Broderick, G.; Lopez-Campistrous, A.; Ru'aini, M.; Winter, P.; Hamilton, M.; Boulanger, P.; Kovalenko, A.; Ellison, M. J. Coarse-Grained Molecular Simulation of Diffusion and Reaction Kinetics in a Crowded Virtual Cytoplasm. *Biophysical Journal* **2008**, *94*, 3748–3759.

(29) Skóra, T.; Janssen, M.; Carlson, A.; Kondrat, S. Crowding-Regulated Binding of Divalent Biomolecules. *Phys. Rev. Lett.* **2023**, *130*, 258401.

(30) Miyaguchi, T. Reduction of Self-Diffusion Coefficient in a Coarse-Grained Model of Cytoplasm. *Phys. Rev. Res.* **2020**, *2*, 013279.

(31) Jeffrey, D. J.; Onishi, Y. Calculation of the Resistance and Mobility Functions for Two Unequal Rigid Spheres in Low-Reynolds-number Flow. *J. Fluid Mech.* **1984**, *139*, 261–290.

(32) Kalwarczyk, T.; Ziebacz, N.; Bielejewska, A.; Zaboklicka, E.; Koynov, K.; Szymański, J.; Wilk, A.; Patkowski, A.; Gapiński, J.; Butt, H. J.; Hołyst, R. Comparative analysis of viscosity of complex liquids and cytoplasm of mammalian cells at the nanoscale. *Nano Lett.* **2011**, *11*, 2157–2163.

(33) Kalwarczyk, T.; Ziębacz, N.; Bielejewska, A.; Zaboklicka, E.; Koynov, K.; Szymański, J.; Wilk, A.; Patkowski, A.; Gapiński, J.; Butt, H.-J.; Hołyst, R. Comparative Analysis of Viscosity of Complex Liquids and Cytoplasm of Mammalian Cells at the Nanoscale. *Nano Lett.* **2011**, *11*, 2157–2163.
